# The Arabidopsis RboHB Encoded by *At1g09090* Is Important for Resistance against Nematodes

**DOI:** 10.3390/ijms21155556

**Published:** 2020-08-03

**Authors:** Abdalmenem I. M. Hawamda, Adil Zahoor, Amjad Abbas, Muhammad Amjad Ali, Holger Bohlmann

**Affiliations:** 1 Institute of Plant Protection, Department of Crop Sciences, University of Natural Resources and Life Sciences, 1180 Vienna, Austria; abdalmenem.hawamda@boku.ac.at (A.I.M.H.); amjad.abbas@uaf.edu.pk (A.A.); 2 Department of Agricultural Biotechnology, Faculty of Agricultural Science and Technology, Palestine Technical University-Kadoorie (PTUK), P.O. Box 7, Tulkarm, Palestine; 3 Department of Biotechnology, Chonnam National University, Yeosu, Chonnam 59626, Korea; adilzahoor3253@gmail.com; 4 Department of Plant Pathology, Faculty of Agriculture, University of Agriculture, Faisalabad 38040, Pakistan; 5 Centre of Agricultural Biochemistry and Biotechnology, University of Agriculture, Faisalabad 38040, Pakistan

**Keywords:** Arabidopsis, *Heterodera schachtii*, plant defense, reactive oxygen species (ROS)

## Abstract

Reactive oxygen species are a byproduct of aerobic metabolic processes but are also produced by plants in defense against pathogens. In addition, they can function as signaling molecules that control various aspects of plant life, ranging from developmental processes to responses to abiotic and biotic stimuli. In plants, reactive oxygen species can be produced by respiratory burst oxidase homologues. Arabidopsis contains 10 genes for respiratory burst oxidase homologues that are involved in different aspects of plant life. Plant pathogenic cyst nematodes such as *Heterodera schachtii* induce a syncytium in the roots of host plants that becomes a feeding site which supplies nutrients throughout the life of the nematode. In line with this function, the transcriptome of the syncytium shows drastic changes. One of the genes that is most strongly downregulated in syncytia codes for respiratory burst oxidase homologue B. This gene is root-specific and we confirm here the downregulation in nematode feeding sites with a promoter::GUS (β-glucuronidase) line. Overexpression of this gene resulted in enhanced resistance against nematodes but also against leaf-infecting pathogens. Thus, respiratory burst oxidase homologue B has a role in resistance. The function of this gene is in contrast to respiratory burst oxidase homologues D and F, which have been found to be needed for full susceptibility of Arabidopsis to *H. schachtii.* However, our bioinformatic analysis did not find differences between these proteins that could account for the opposed function in the interaction with nematodes.

## 1. Introduction

Reactive oxygen species (ROS) [1,2] such as hydrogen peroxide (H_2_O_2_), superoxide (O_2_^−^) and singlet oxygen (^1^O_2_) are unavoidable byproducts of aerobic metabolic processes. They are highly reactive and could cause oxidative damage to DNA, proteins and other molecules of the cell. There are different cellular mechanisms in place to deactivate these damaging ROS molecules. These include enzymatic reactions through catalase, superoxide dismutase, glutathione peroxidase and ascorbate peroxidase [1] but also small antioxidants such as ascorbic acid and glutathione [3]. In addition to these metabolic byproducts, plants also use ROS directly as a defense against pathogens [4]. In this case, ROS are produced by oxidases and peroxidases to generate an apoplastic oxidative burst which could directly kill pathogens [5,6,7,8]. After a first, rapid phase of ROS production, a second, sustained phase can occur, which is associated with the hypersensitive response [9].

Another important aspect of ROS in plants is their use as signaling molecules (reviewed by [2,4,10,11]). ROS control various aspects of plant life, ranging from developmental processes to responses to abiotic and biotic stimuli. A key group of genes involved in the production and signaling of ROS in plants code for respiratory burst oxidase homologues (Rboh). They contain a conserved C-terminal core region of six transmembrane α-helices with two heme groups and an oxidase domain responsible for the generation of superoxide. The N-terminal region contains regulatory domains. Some examples for the roles of ROS include a function of Arabidopsis *RbohB* in seed after ripening [12]. ROS produced by Rboh is also involved in regulating pollen tube growth [13]. In *Medicago truncatula, RbohA* was found to be important for symbiotic nodules induced by *Sinorhizobium meliloti* [14]. Arabidopsis *RbohD* and *RbohF* are involved in ABA signaling as well as in the plant defense response to pathogens [15,16].

Plant pathogenic nematodes are a global threat to agriculture. Cyst nematodes [17] are a group of endoparasitic nematodes that can survive as cysts in the soil for many years. Juveniles hatch from the cyst under favorable conditions to invade the roots of host plants. There, they select an initial syncytial cell to induce the development of a syncytium, which grows by incorporating neighboring cells through local cell wall dissolution. The syncytium is the only source of nutrients for the nematodes throughout their life. Nutrients are taken up through the stylet with the help of feeding tubes that are produced by the nematode in each feeding cycle. To cope with the constant retrieval of nutrients, the syncytium is metabolically highly active, as seen by the degradation of the central vacuole, dense cytoplasm and enlarged nuclei due to endoreduplication of DNA. Female nematodes stay attached to their syncytium for their whole life, while males leave their syncytium after some time in order to mate with females. The females grow to a lemon shape and their body eventually hardens to form a cyst that contains several hundred eggs [18].

Siddique et al. [19] reported that *RbohD* and *RbohF* genes were important for the susceptibility of Arabidopsis plants infected by the beet cyst nematode *Heterodera schachtii*. Mutants for *RbohD* and *RbohF* exhibited larger cell death regions when infected by *H. schachtii*. Mutants also supported a lower number of female nematodes and these were also smaller.

The transcriptome of syncytia induced by *H. schachtii* in Arabidopsis roots reflects its specific function with thousands of upregulated and downregulated genes as compared to control root cells [20]. One gene which is strongly downregulated is *RbohB*, indicating that its expression may be negatively affecting the function of the syncytium or the nematode itself. Here, we have studied the expression and function of *RbohB* and show that this gene has the opposite effect compared to *RbohD* and *RbohF* and can enhance the resistance against plant pathogenic nematodes but also other plant pathogens if expressed in aerial parts of the plant. Given the different functions of *RbohB* and *RbohD*/*RbohF* in response to nematodes, we have included a bioinformatics analysis of all *Rboh* genes and proteins. We found no differences in the Rboh proteins that might explain the different functions.

## 2. Results

### 2.1. Expression of RbohB

**Figure 1:**
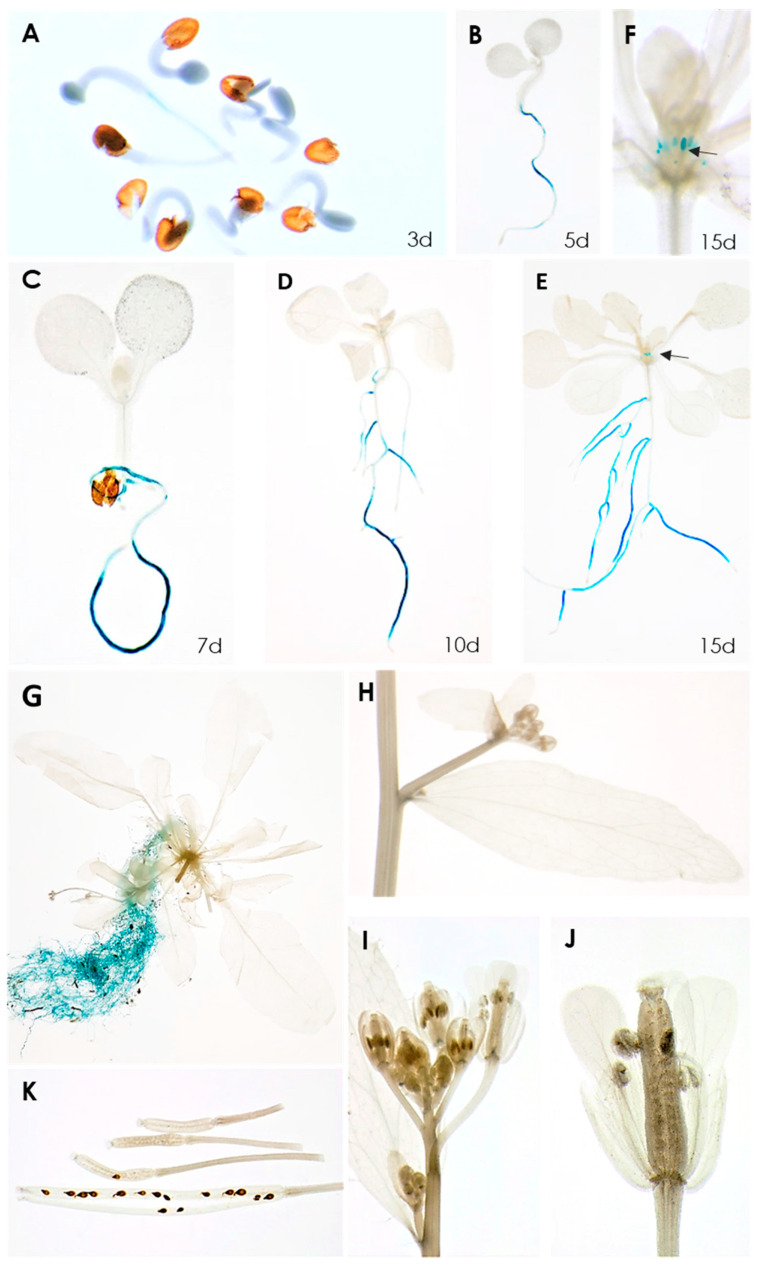
GUS expression in RbohB::GUS transgenic line. (**A**–**E**), seedlings grown on MS medium for 3, 5, 7, 10, 15 days. GUS expression is only visible in roots, except for 15-day-old seedling shown in (**E**). (**F**), Enlargement of 15-day-old seedling showing GUS expression in leaf primordia. (**G**–**K**), plants grown on soil in long day. (**G**), 6-week-old plant showing GUS expression in roots. No GUS expression was found in cauline leaves (**H**), flowers (**I**,**J**) or siliques and seeds (**K**). Arrows in (**E**,**F**) point to leaf primordia.

A transcriptome analysis of syncytia induced by the beet cyst nematode *H. schachtii* in Arabidopsis roots found that, among the 10 *Rboh* genes, seven were significantly downregulated [20]. Of these, *RbohB* was the most strongly downregulated (Table S1), indicating that its expression might have a negative effect on the development of syncytia or the nematodes itself. We have therefore studied this gene in detail. A promoter::GUS analysis showed that its expression was restricted to the roots, starting at around 3 days after germination, with one exception (Figure 1). There was no expression in cotyledons and leaves of different ages; however, we found weak expression in leaf primordia in older seedlings. No GUS expression was found in cauline leaves, stems, flowers and siliques or seeds.

**Figure 2:**
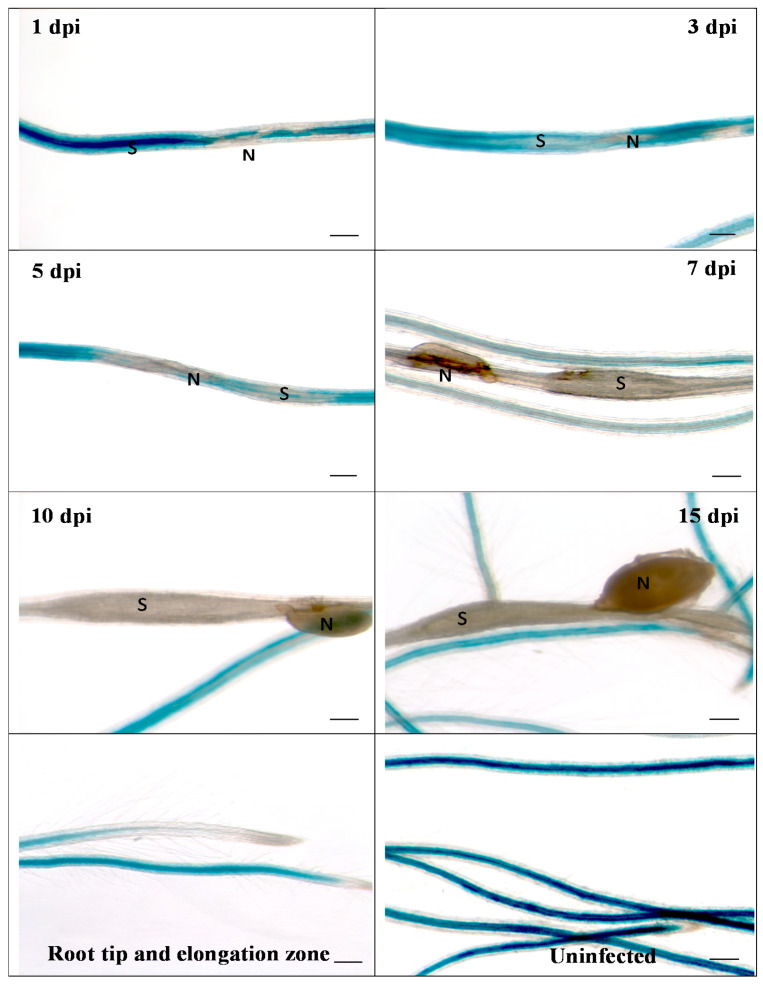
GUS expression in RbohB::GUS transgenic line infected with *H. schachtii*. Shown are infection sites at 1, 3, 5, 7, 10 and 15 dpi of roots grown on Knop medium as well as uninfected roots and root tips with elongation zone. Downregulation of GUS expression at infection sites starts as early as 1 dpi. S, syncytium; N, nematode. Bar = 100 μm.

**Figure 3:**
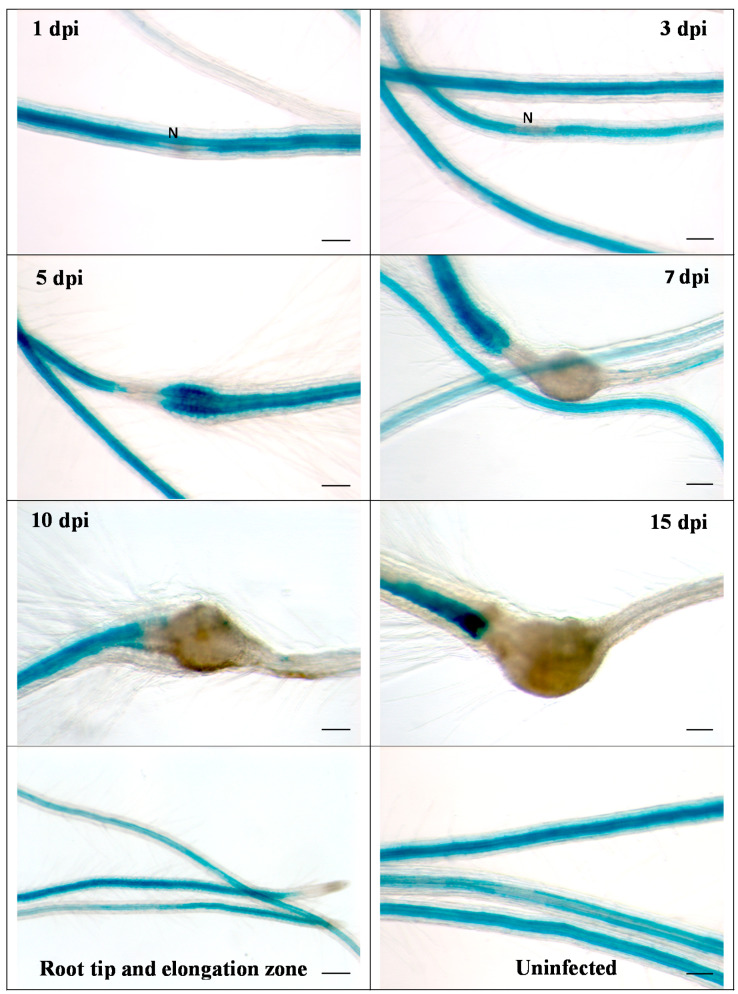
GUS expression in RbohB::GUS transgenic line infected with *M. incognita*. Shown are infection sites at 1, 3, 5, 7, 10 and 15 dpi of roots grown on Knop medium as well as uninfected roots and root tips with elongation zone. Downregulation of GUS expression at infection sites is clearly visible at 5 dpi. There was no GUS expression in galls at 7, 10 and 15 dpi. N, nematode. Bar = 100 μm.

We then infected the promoter::GUS line with second stage juveniles of *H. schachtii* (Figure 2). Already at 1 dpi, a downregulation of GUS at the infection site was visible, which became more pronounced during later stages of nematode development. Thus, these data confirmed the downregulation of *RbohB* from transcriptome data. The same picture emerged after infection of the promoter::GUS line with *M. incognita*. There was again a downregulation of GUS expression, clearly visible at 5 dpi (Figure 3). There was no staining in galls from 7 dpi onwards.

The downregulation of *RbohB* induced by plant pathogenic nematodes indicates a possible function of *RbohB* in resistance. Although we did not find expression in leaves, one might thus assume that *RbohB* could be induced in leaves by infection with plant pathogens. We therefore infected the *RbohB* promoter::GUS line with *Alternaria brassicicola*, *Botrytis cinerea* and *Pseudomonas syringae* pv *tomato* DC3000. There was no GUS visible after staining at different time points after infection (Figure S1).

### 2.2. Overexpression of RbohB Results in Enhanced Resistance against Nematodes

**Figure 4:**
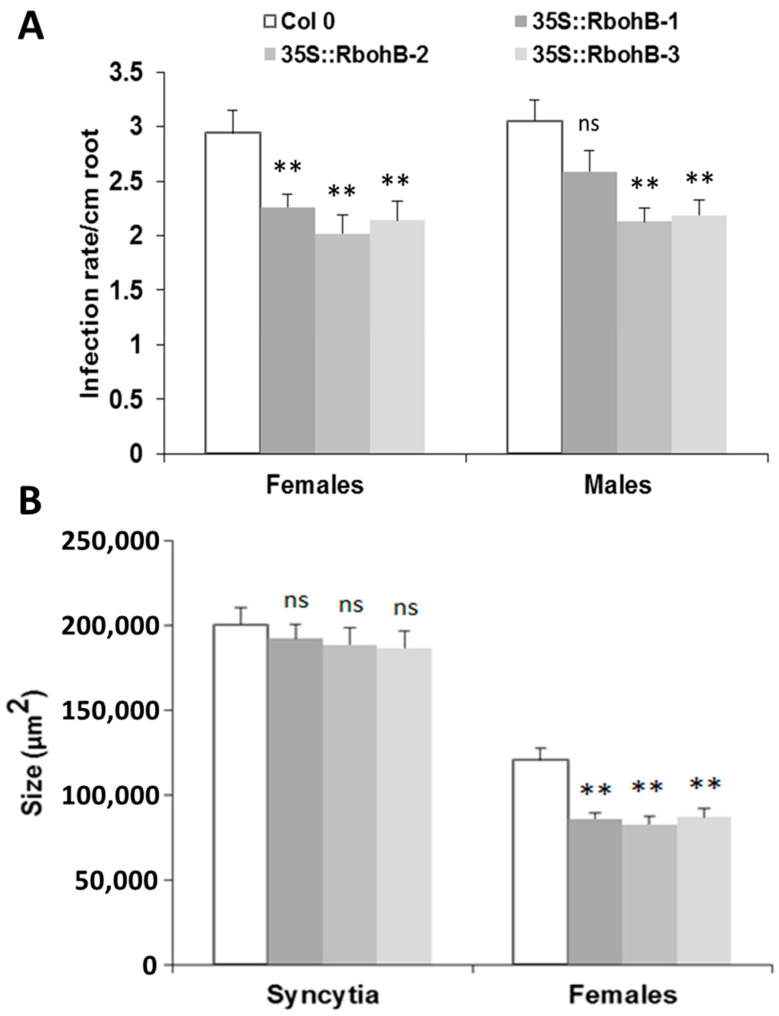
*H. schachtii* resistance test. The resistance of overexpression lines of *RbohB* was compared to wild type plants after infection with *H. schachtii*. (**A**), Number of male and female nematodes per cm of root length, calculated at 15 dpi. Infection rate is shown in column sets, with asterisks indicating significant differences (** *p* < 0.05; ANOVA and LSD). The statistical significance was determined by three independent replicates. Values are means ± SE, *n* = 15. The bar shows standard error for the mean. Ns, not significant (**B**), Size of female syncytia and female nematodes at 14 dpi. Five syncytia were selected randomly from three independent replicates (total = 15) and the size of syncytia and associated female nematodes were measured. Data were analyzed for significance difference using ANOVA (*p* < 0.05) and LSD. Values are means ± SE.

The downregulation in feeding sites of *H. schachtii* and *M. incognita* indicated that expression of *RbohB* might affect the susceptibility of Arabidopsis roots to these pathogenic nematodes. We therefore produced overexpression lines for *RbohB* and tested them for resistance against both nematode species. Seedlings of three independent *RbohB* overexpression lines and a wild type were infected with *H. schachtii* larvae. All three overexpression lines supported a significantly lower number of female nematodes than the wild type, while the number of male nematodes was reduced in two of the three overexpression lines (Figure 4A). The size of syncytia was not affected by the overexpression of *RbohB* but the size of female nematodes was significantly smaller on all three overexpression lines as compared to the wild type (Figure 4B).

**Figure 5:**
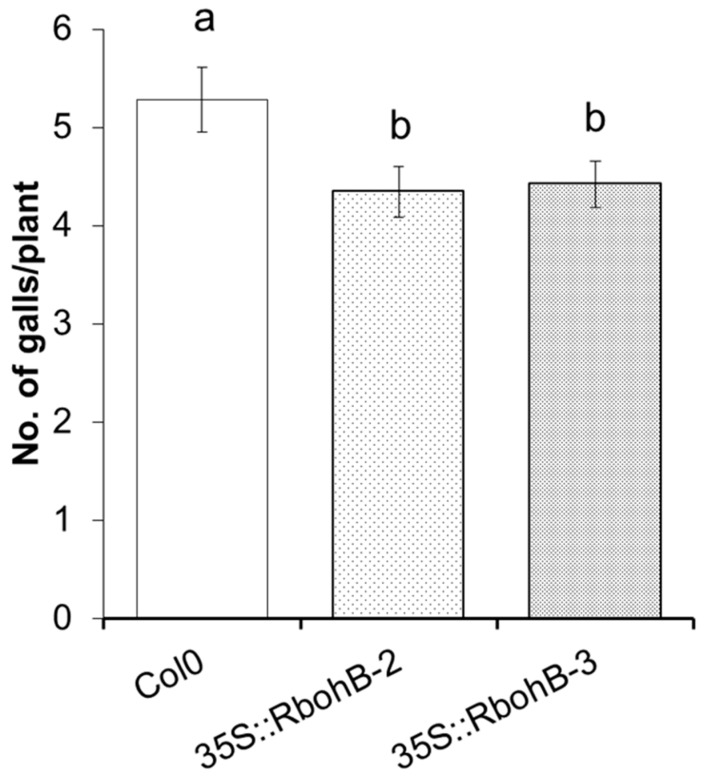
*M. incognita* resistance test. The resistance of two overexpression lines of *RbohB* was compared to wild type plants after infection with *M. incognita*. Number of galls per roots were counted at 15 dpi with different letters “a” and “b” indicating significant difference (*p* < 0.05; ANOVA and LSD). The statistical significance was determined by three independent replicates. Values are means ± SE, *n* = 12.

We also tested two of the overexpression lines with *M. incognita*. The number of galls was counted at 15 dpi and was approximately 20% lower on the roots of overexpression lines as compared to the wild type (Figure 5).

**Figure 6:**
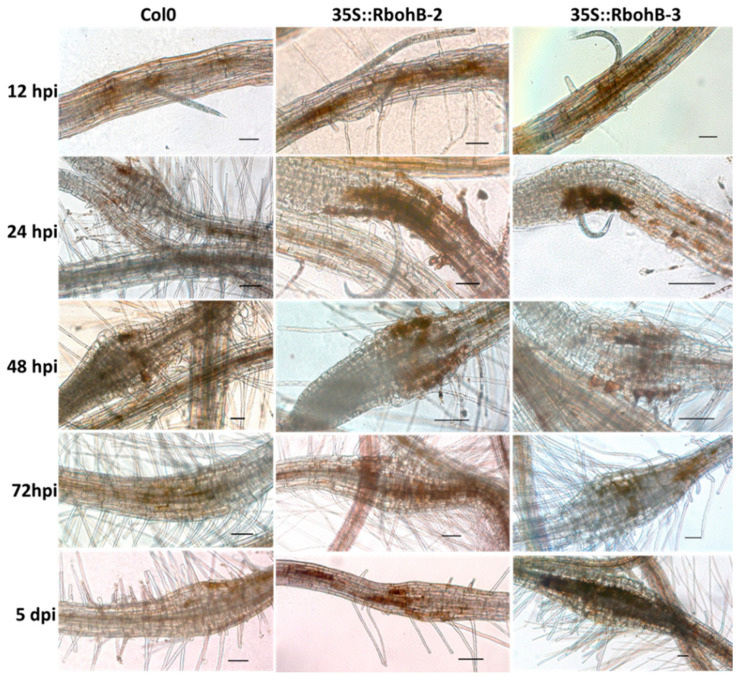
Reactive oxygen species (ROS) production. DAB (3, 3-diaminobenzidine) staining of infection sites of *M. incognita* in wild type and overexpression lines of *RbohB* at different time points after inoculation. Scale bar = 100 µm.

Rboh proteins are involved in the production of ROS. We tested the ROS production in roots after infection with *M. incognita* larvae (Figure 6). DAB staining showed that there was enhanced ROS production in the two overexpression lines. Since the size of syncytia in the roots of overexpression lines was not significantly different from those in wild type roots, this could indicate that the enhanced resistance of *RbohB* overexpression lines is due to a direct effect on the nematodes.

### 2.3. Overexpression of RbohB Results in Enhanced Resistance against Leaf-Infecting Pathogens

**Figure 7:**
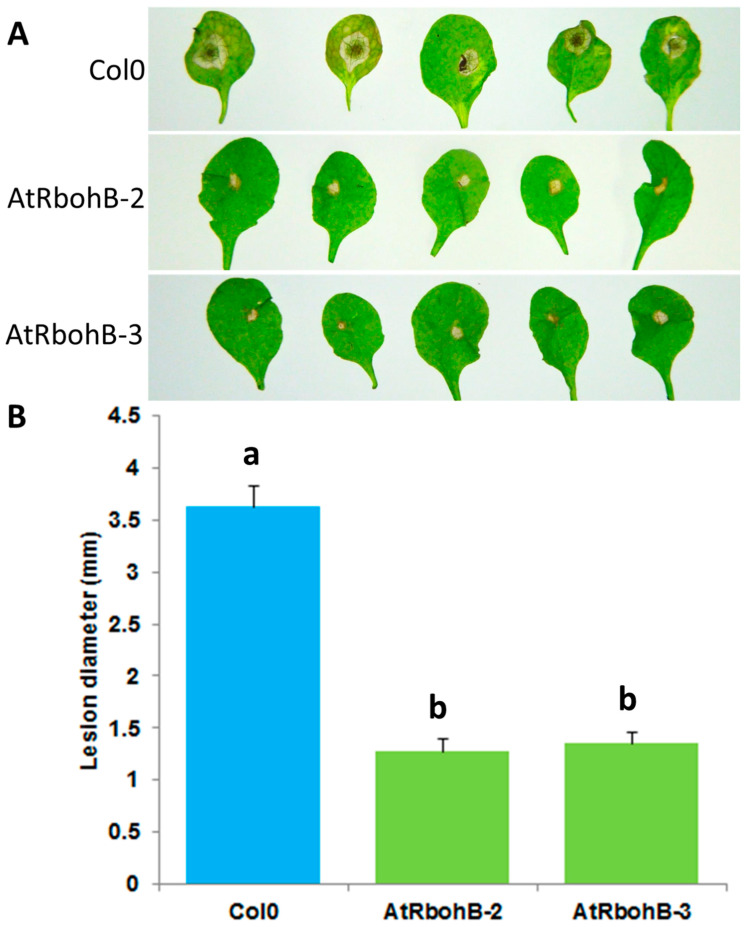
Functional analysis of *RbohB* overexpression lines in response to *B. cinerea* infection. (**A**): *B. cinerea* infection test to evaluate the involvement of *RbohB* in plant defense. Overexpression lines of *RbohB* were compared with wild type plant after infection. Plants were infected with *B. cinerea* by putting a 5 μL drop on the surface of 4-week-old leaves. Plants were grown in short day conditions with 16 h dark and 8 h light at 24 °C. Representative pictures were taken at 5 dpi. (**B**): Lesion diameter in mm was determined at 5 dpi. For each line in a replicate, three plants were infected. Mean data from three experimental replications were subjected to analysis of variance and differences among mean determined by LSD at 5 % significance level and *n* = 15 with different letters “a” and “b” indicating significant difference. Values are means ± SE.

**Figure 8:**
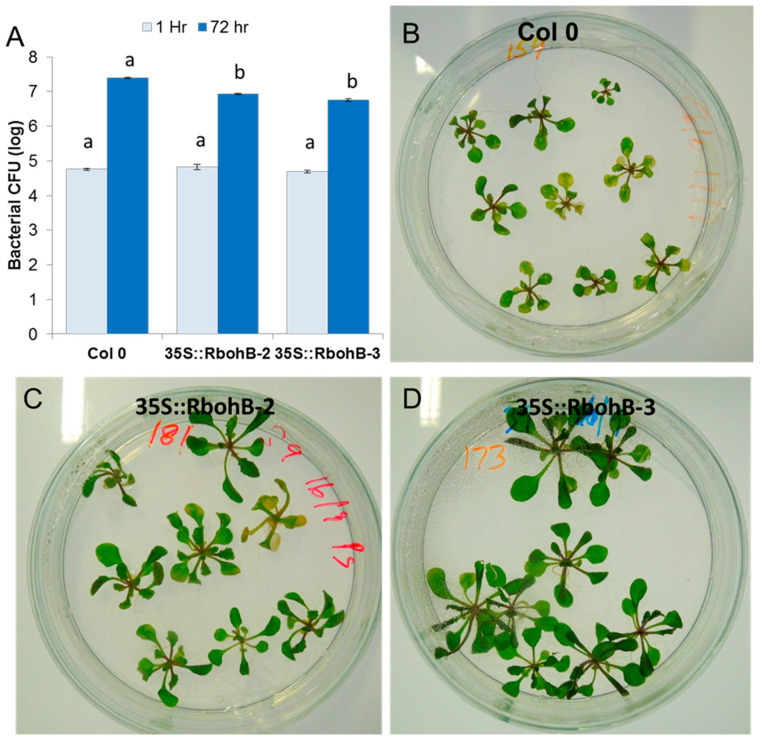
Functional analysis of *RbohB* overexpression lines in response to *P. syringae* pv *tomato* DC3000. (**A**), Overexpression lines and wild type control were infected in plates by the flooding method. Four independent biological replicates were used to collect the data. Data were analyzed for significance difference using ANOVA (*p* < 0.05) and LSD with different letters “a” and “b” indicating significant difference. Values are means ± SE. (**B**–**D**): Phenotypic response of the overexpression lines showed less chlorosis and necrosis at 3 dpi as compared to control wild type.

Our expression analysis has confirmed that *RbohB* is not expressed in leaves (with the exception of leaf primordia). However, the expression of *RbohB* in our overexpression construct was driven by the 35S CaMV (cauliflower mosaic virus) promoter, which is also expressed in leaves. Since other *Rboh* genes are involved in resistance against pathogens in the green part of the Arabidopsis plant [11], we tested whether the overexpression of *RbohB* would also lead to enhanced resistance against bacteria or fungi. Infection of the leaves with *Botrytis cinerea* spores resulted in much smaller lesions on the overexpression lines as compared to wild type plants (Figure 7). The overexpression lines were also more resistant to *Pseudomonas syringae* (Figure 8), supporting approximately five times less bacteria after 3 days. There was also a phenotypic difference between the overexpression lines and the wild type after infection with *P. syringae*. The seedlings of the overexpression lines were much larger after infection and also of a darker green color.

### 2.4. In Silico Characterization of Arabidopsis Rboh Genes and Proteins

Our results showed that *RbohB* is involved in resistance against plant pathogenic nematodes. On the other hand, it has been reported that *RbohD* and *RbohF* are needed for full susceptibility of Arabidopsis to *H. schachtii* [19]. We have therefore conducted a bioinformatic analysis of the Arabidopsis *Rboh* genes to explore whether it might reveal differences between these genes/proteins that could explain their different behaviors in the interaction with nematodes.

**Figure 9:**
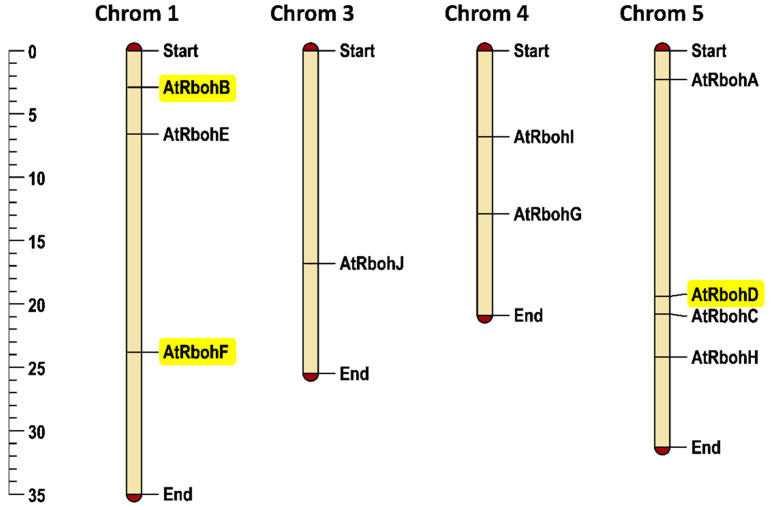
Chromosomal positioning of Arabidopsis *Rboh* genes. Scale provided on left side of the figure shows chromosomal length in mega base pairs (mbp). Genes of special interest for this work are highlighted in yellow. Each chromosome is labelled with its corresponding chromosomal number at the top of the figure.

Arabidopsis has 10 *Rboh* genes, with three genes on chromosome 1 and one gene on chromosome 3. No *Rboh* gene was found on chromosome 2, while two and four *Rboh* genes were mapped on chromosomes 4 and 5, respectively. *RbohB* and *RbohF* are located on chromosome 1 while *RbohD* was found on chromosome 5. In general, the chromosomal positioning of *Rboh* genes (Figure 9) represented uneven distribution in the Arabidopsis genome.

The genomic DNA lengths of all *Rboh* genes varied from 3848 *(RbohB)* to 7211 (*RbohF*) base pairs. Similarly, coding sequence length ranged from 2535 to 2859 base pairs and protein length ranged accordingly from 843 to 952 amino acids. The molecular weight of all Rboh proteins varied from 96,389.3 to 108,417.3 Daltons. RbohI exhibited the lowest isoelectric point of 8.58 whereas RbohC had the highest isoelectric point of 9.91. The lowest and highest GRAVY (grand average of hydropathy) to be calculated were −0.156 (RbohB) and −0.287 (RbohF), respectively. No signal peptide cleavage site was found in any of the Rboh protein sequences (Table S2).

**Figure 10:**
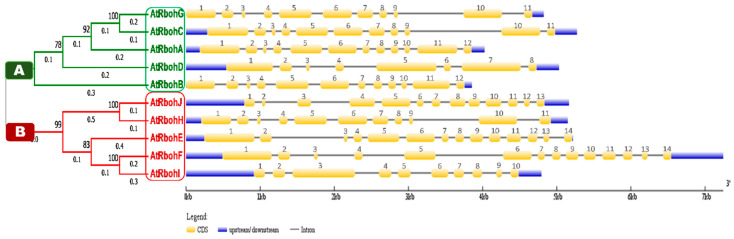
Phylogenetic analysis in line with intron-exon structure analysis of Arabidopsis *Rboh* genes. Phylogenetic tree is classified into group A and B, which are labeled by green and red boxes, respectively. Scale provided at the bottom of the figure compares the length of gene in 1000 base pairs (kbp). Golden, blue and thin grey colored lines show positions of exons, UTRs and introns, respectively.

We conducted a phylogenetic analysis by using the Rboh protein sequences, which resulted in a tree with two equal groups, A and B (Figure 10), containing five genes each. *RbohG, RbohC, RbohA, RbohD* and *RbohB* were clustered into group A while *RbohJ, RbohH, RbohE, RbohF* and *RbohI* were found in group B.

Gene structure analysis of all 10 *Rboh* genes was performed to compare the intron-exon patterns in their genomic DNA. Members of group A and B showed various similarities in size and relevant positions of introns, exons and untranslated regions (UTRs). For instance, *RbohG* and *RbohC* genes from group A and *RbohH* from group B presented 11 exons of similar size and relevant positions (Figure 10). Similarly, *RbohA* and *RbohB* of group A had 12 exons of similar size and relevant prevalence in genomic DNA. We found 14 exons of similar size in both *RbohE* and *RbohF* (group B); however, likely due to intronic insertions (*RbohF)* or deletions (*RbohE*)*,* the relevant positions of 14 exons were different in both genes. The lowest number of eight exons was found in *RbohD*. No 5′UTRs were found for the group A genes *RbohG* and *RbohB*.

### 2.5. Conserved Motif and Domain Analysis of Rboh Proteins

**Figure 11:**
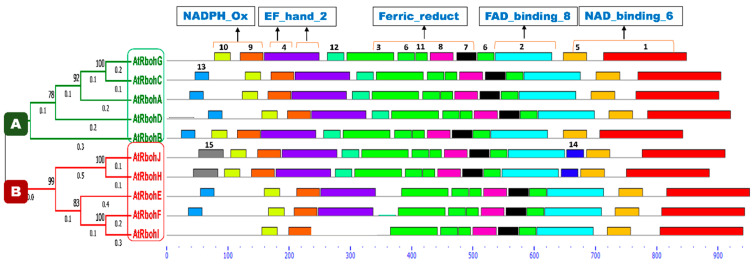
Conserved motif analyses of Rboh proteins. Phylogenetic tree is classified into group A and B, which are labeled with green and red color boxes, respectively. Motif numbers from 1 to 15 with corresponding colors (see also Figure S2) assigned by the MEME suite tool are indicated at top of each motif. Motifs’ heights depict the significance of match, whereas motifs’ widths display the number of amino acids in the respective motif. Characteristic domains corresponding to motifs are indicated at the top of the figure. Rboh proteins containing EF_hand_2 motifs are highlighted with orange lines on motif 4. The *X*-axis shows the number of amino acids.

A conserved motif analysis found a total of 15 different motifs (Figure 11). All members of both groups A and B were common in the presence of motifs 1, 2, 3, 5, 6, 7, 8, 9, 10 and 11, while prevalence of all other motifs was not found in any sequences of either group. Motif 4 was found in all members except RbohI, while motif 12 was present in group A and, in addition, in RbohJ and RbohH from group B. An uneven distribution between both groups was detected for motif 13. A partial difference that could be found between groups A and B was the presence of motifs 14 and 15, occurring only in RbohJ and RbohH of group B. All predicted motifs’ widths, sites, E-values, consensus sequences and sequence logos are provided in Table S3 and Figure S2, respectively.

A conserved domain analysis for the occurrence of different characteristic domains in Arabidopsis Rboh proteins was performed using the InterPro online database [21]. As a result, four domains—namely NADPH_Ox, Ferric_reduct, FAD_binding_8 and NAD_binding_6—were found in all 10 Rboh proteins (Figure S4). In addition, the EF_hand_2 motif for Ca^2+^ binding was found at the N-terminus in motif 4, in close proximity to the NADPH_Ox domain, in all Rboh proteins except RbohI, in which motif 4 is missing. RbohA, RbohJ, RbohE and RbohF showed one EF_hand_2 motif while the other Rboh proteins, except RbohI, displayed this motif twice. Several transmembrane regions which anchor the protein to the plasma membrane were also detected in all proteins. These regions were mainly predicted at the start, in the middle and at the end of the Ferric_reduct domain in all polypeptides (Figure S3). In general, the domain architecture of all Rboh proteins is very similar (Figure S4) and could not explain the different functions of RbohB and RbohD/RbohF in relation to *H. schachtii*.

The majority of conserved motifs corresponded to characteristic domains of Rboh family proteins. Motifs 9 and 10 were located in the region of the NADPH_Ox domain in all proteins and EF_hand_2 motif was represented by motif 4. Motifs 3, 6, 7, 8 and 11 were found in the Ferric_reductase domain. FAD_binding_8 and NAD_binding_6 domains were represented by motifs 2 and 1, respectively. Transmembrane regions (TM) were mostly found at the N-terminus of motif 3.

Our comparison of motifs and domains did not reveal a major difference between RbohB and RbohD/RbohF. We therefore aligned the protein sequences for these (Figure S5). This clearly showed major differences at the N-terminus, with several larger insertions/deletions. RbohD had three small insertions at the N-terminus compared to RbohB and RbohF. Following these are an insertion in RbohD and RbohF and two insertions in RbohF compared to RbohB and RbohD. There were only small differences in the remaining parts of the proteins, mostly single amino acid changes. The only exception was a 10 amino acid insertion in RbohF, approximately 150 amino acids from the C-terminus.

## 3. Discussion

### 3.1. Expression of RbohB

Our expression analysis using a promoter::GUS line found that *RbohB* is expressed in roots, which is confirmed by data from Genevestigator [22], as shown in Figure S6. The only exception that we found was expression in leaf primordia. The expression in roots was downregulated in feeding sites induced by plant pathogenic nematodes *H. schachtii* and *M. incognita*. Data from a transcriptome analysis of syncytia induced by *H. schachtii* [20] confirm this and show in addition that most *Rboh* genes are actually downregulated in syncytia. Downregulation in response to infection with *M. incognita* is confirmed by data from Genevestigator (Figure S7). There is no indication that pathogen infection of Arabidopsis leaves leads to induction of *RbohB* (Figure S7) and infection of the promoter::GUS line with *A. brassicicola*, *B. cinerea* and *P. syringae* also did not result in any GUS expression, as revealed by staining.

There is currently only one report about Arabidopsis *RbohB* [12]. The authors reported a role in seed after ripening by comparing an *RbohB* mutant with a wild type. They found that Arabidopsis RbohB is a major producer of ROS in germinating seeds. Accordingly, they detected the *RbohB* mRNA in dry seeds and 24 h imbibed seeds using RT-PCR; however, the band from dry seeds was very weak. This might explain why we did not detect expression in seeds with our GUS line. The expression in imbibed seeds is probably due to expression in the emerging root.

Compared to *RbohB*, *RbohD* and to a lesser extent *RbohF* were not only expressed in roots but also in aerial tissues. In line with a role in plant immunity, *RbohD* was also induced by different PAMPs (pathogen-associated molecular patterns), flagellin, ELF18 and chitin. Both genes were also induced, although at different levels and in different tissues, after inoculating the leaves with pathogenic bacteria and fungi [23]. For *RbohB*, there are only GeneChip data available which show that this gene is not induced in response to plant pathogenic bacteria and fungi (Figure S7), as we have also shown here. In response to PAMPs, there might be a very weak induction in response to flagellin and Pep2, but this has to be confirmed (Figure S8). Thus, *RbohB* and *RbohD*/*RbohF* respond differently in response to plant pathogenic bacteria and fungi as well as to plant pathogenic nematodes. In the case of the latter, while *RbohB* is strongly downregulated in syncytia induced by *H. schachtii*, *RbohD* expression remains at a high level and *RbohF* is only slightly downregulated (Table S1) [20].

### 3.2. Enhanced Resistance of RbohB Overexpression Lines

The strong downregulation of *RbohB* in syncytia indicated that *RbohB* expression might be detrimental to nematode development. We tested this with overexpression lines. We used the CaMV promoter to drive expression of *RbohB* because this promoter is active in most plant tissues. A disadvantage of the CaMV promoter is the downregulation in syncytia [24,25]. However, it is still active in the early stages of syncytium development and the possibility to test also against leaf infecting pathogens outweighs this disadvantage [26].

We tested three independent overexpression lines with *H. schachtii*. All three lines supported significantly less female nematodes compared to the wild type, and two of them also supported significantly less male nematodes. The size of syncytia developing on all three overexpression lines seemed to be smaller but the effect was not significant. On the other hand, the size of female nematodes on all three overexpression lines was significantly smaller than on wild type roots. Thus, the overexpression of RbohB might have a direct effect on the nematodes through the production of ROS. This is supported by the enhanced ROS production that we found on the overexpression lines infected with *M. incognita*. A *RbohB* mutant was recently tested by Siddique et al. [19]. They found that there was no significant difference between the mutant and wild type in the number of nematodes at 14 dpi. There was also no difference in the size of the female nematodes and the size of syncytia induced by female nematodes. The reason that the mutant did not show an effect is that the expression of *RbohB* in syncytia, as shown here and by Szakasits et al. [20], is fast and strongly downregulated.

We also infected two of the overexpression lines with *M. incognita*. On both overexpression lines, the number of galls was significantly reduced compared to the wild type. In addition to the root infecting nematodes, we tested also leaf infecting pathogens. Although there is no evidence that *RbohB* might be expressed in leaves in response to pathogen infection, the overexpression lines were also more resistant to *B. cinerea* and *P. syringae*. Thus, RbohB has an effect against different kinds of plant pathogens. Although our data indicate a direct effect of the produced ROS, we also cannot exclude that there is an indirect effect via ROS acting as signal for the induction of, for instance, PR genes [27]. A transcriptome analysis of the overexpression lines might indicate whether this is the case.

### 3.3. Opposite Effects of RbohB and RbohD/RbohF on H. schachtii

Our results clearly show that *RbohB* is downregulated in syncytia to suppress the negative effect on syncytium development, as shown by overexpression. On the other hand, it has been shown that *RbohD* and to a lesser extent *RbohF* are needed for full susceptibility of Arabidopsis plants to *H. schachtii* [19]. How can these opposing effects be explained? We compared the *Rboh* genes and proteins. A phylogenetic analysis placed the *Rboh* genes in two groups, having *RbohB* and *RbohD* in group A and *RbohF* in group B. Our bioinformatics analysis of the Rboh proteins found that all 10 Arabidopsis proteins are very similar. All contain the same set of functional domains. There are small differences in the transmembrane domains and the EF_hand-2 domains. However, RbohB and RbohD both have two EF_hand-2 domains while RbohF has only one. Thus, this analysis could not explain the different effects on *H. schachtii*. We then aligned the protein sequences of RbohB, RbohD and RbohF (Figure S5). This clearly showed major differences (insertions/deletions) at the N-terminus, but whether these can explain the different functions is not clear. It would be necessary to test this with domain swap experiments.

Given that there are only small differences between all Arabidopsis Rboh proteins, the different functions might be explained by the different expression levels of the *Rboh* genes. However, this cannot explain the different functions of *RbohB* and *RbohD*/*RbohF* on *H. schachtii*. Expression of *RbohB* decreases susceptibility while expression of *RbohD* and *RbohF* increases susceptibility to *H. schachtii* infection. This leaves the possibility that these Rboh proteins cooperate with different interaction partners to achieve their different functions. Indeed, a recent in silico analysis of the Arabidopsis *Rboh* genes [28] has indicated different pathways with different interacting partners, which could explain the different functions of RbohB and RbohD/F in interaction with *H. schachtii*. RbohD and RbohF together with RbohA share a set of six common functional partners, all of them kinases, which are not involved with any of the other Rboh proteins. In line with the large number of potentially interacting kinases, there was a large number of potential phosphorylation sites identified, most of them in RbohD. Moreover, most potential phosphorylation sites are located at the N-terminus. Given the differences in protein sequence at the N-terminus, phosphorylation of RbohD and RbohF could explain the importance of susceptibility against *H. schachtii* in contrast to RbohB. While nothing is known about *RbohA*, which is downregulated in syncytia (Table S1), *RbohD* and *RbohF* are for instance both involved in pathogen defense [15,29], jasmonic acid induced gene expression [30], salinity stress response [16] and stomatal closure [31]. Furthermore, as has already been mentioned, *RbohD* and *RbohF* are both important for susceptibility to *H. schachtii* [19].

The remaining *Rboh* genes seem to have specific and different functions since they have mostly specific interaction partners [28]. No function has been reported for *RbohG*. *RbohB* is involved in seed after-ripening [12], *RbohC* in root hair tip growth [32], *RbohE* in tapetum cell death [33] and *RbohI* in drought stress response in seeds and roots [34]. While the aforementioned genes have specific functions, which might be explained by their specific interaction partners, two other genes, *RbohH* and *RbohJ*, have a redundant function. Both are involved in polar root hair growth and pollen tube tip growth [35] while having only one common interacting partner, which, however, is also found as the interacting partner for several other *Rboh* genes, including *RbohB*.

While RbohD and RbohF share this set of potential kinase partners, the bioinformatics analysis of Kaur and Pati [28] identified only one specific interaction partner for each of them. Contrastingly, for RbohB, there were five unique functional partners that were not found for any of the other Rboh proteins. They all have different functions and none of them are kinases. However, the CPK6 kinase was identified as a functional partner for RbohB, RbohD and RbohF. Whether one or more of the unique functional partners of RbohB might be responsible for the resistance function of RbohB is not known and would have to be tested experimentally.

It could also be the case that the activity of RbohB and RbohD/RbohF leads to different ROS species, which could result in the observed effects on nematode interaction. However, to our knowledge, nothing is known about this possibility. Kaya et al. [36] have studied the ROS-producing activity of different Arabidopsis Rboh enzymes in a bacterial expression system. They found that RbohB had the lowest activity while RbohH and RbohJ had the highest activity, about 100-fold that of RbohB. RbohD and RbohF were somewhere in the middle. Whether these differences in activity between RbohB on the one side and RbohD and RbohF on the other side can account for the different behavior in response to *H. schachtii* is not clear. It has also been taken into account that all Arabidopsis Rboh enzymes are activated by phosphorylation [37].

## 4. Materials and Methods

### 4.1. Cloning of Binary Vectors

We used the vector pMAA-Red [38] for overexpression analysis and GUS analysis. For cloning of the overexpression vector, the *RbohB* coding sequence was amplified from cDNA by PCR. The internal NcoI site was eliminated as follows. The coding sequence was amplified in two parts using primers At1g09090forNco and At1g09090Mrev and At1g09090Mfor and At1g09090revBam, respectively, containing the restriction sites NcoI and BamHI (Table S4). An overlap PCR was then done with both fragments and primers At1g09090forNco and At1g09090revBam. The final PCR fragment was digested with NcoI and BamHI and ligated into the vector pMAA-Red, digested with the same enzymes. The final construct was confirmed by sequencing.

For cloning of a promoter::GUS fusion, we amplified the promoter region by PCR, using as a template Arabidopsis genomic DNA. Primers pRBOHBforKpn and pRBOHBrevNco (Table S4) included restriction sites for KpnI and NcoI, respectively. The PCR fragment was digested with KpnI and NcoI and ligated to the large vector fragment of pMAA-Red, digested with the same enzymes, thus replacing the 35S promoter with the *RbohB* promoter. The construct was verified by sequencing.

### 4.2. Plant Material and Growth Conditions

Arabidopsis seeds (ecotype Columbia) were surface sterilized for 20 min in 6% (*w*/*v*) sodium hypochlorite and subsequently washed three times with sterile water for in vitro growth on either Murashige and Skoog (MS) medium or Knop medium [39]. For seed production, Arabidopsis plants were cultivated on soil in a growth chamber at 25 °C in a 16 h light and 8 h dark cycle.

### 4.3. Arabidopsis Transformation

Binary vectors were introduced into *Agrobacterium tumefaciens* GV3101 by a freeze-thaw method [40] for transformation of *Arabidopsis* plants by a modified floral dip method [41]. Transformed seeds were identified as described by [38] and transferred to soil for seed production.

For the promoter::GUS construct, 12 independent transgenic plants were generated and tested for GUS activity to choose a representative line, which was grown further to produce homozygous seeds. For overexpression lines, 12 independent transgenic T2 lines were generated and applied to RT-PCR using the primers described in Table S4 to select the best expressing lines. These were then made homozygous for resistance tests.

### 4.4. Resistance Tests with H. schachtii and M. incognita

*H. schachtii* was multiplied on mustard (*Sinapis alba* cv. Albatros) roots in vitro under sterile conditions. Cysts were collected from the mustard stock cultures and J2 larvae were hatched by soaking the cysts in 3 mM ZnCl_2_. Larvae were washed three times in sterile water. They were resuspended in 0.5% (*w*/*v*) gelrite (Duchefa, Haarlem, The Netherlands) for infection of Arabidopsis roots.

Arabidopsis seedlings were grown on 0.2 concentrated Knop medium supplemented with 2% sucrose [39] for 12 days. Roots were then inoculated with approximately 50–60 J2 larvae per plant. Three independent experiments were performed with 5 Petri dishes each, with one Petri dish containing approximately 10 seedlings. Female and male nematodes were counted at 14 dpi and the number of males and females per cm of root length was calculated. At 15 dpi, female nematodes and syncytia associated with female nematodes were photographed using an inverse microscope (Axiovert 200M; Zeiss, Hallerbergmoos, Germany) with an integrated camera (AxioCam MRc5; Zeiss, Hallerbergmoos, Germany) and measured according to [42].

*M. incognita* egg masses were harvested from sterile stock cultures propagated on cucumber roots which were grown on B5 agar medium. The hatching of second-stage juveniles (J_2_) of *M. incognita* was stimulated by soaking the egg masses in sterile water for 2–4 days at room temperature in the dark. Freshly hatched J_2_ larvae were sterilized by incubation in sterilization solution (0.002% HgCl_2_) for 5–10 min, followed by 3 to 4 washings with sterile distilled water. After sterilization, the J_2_ larvae were suspended in 0.7% gelrite and 12-days-old Arabidopsis plants growing on Knop medium were inoculated with these larvae. The number of galls was counted at 14 dpi.

### 4.5. Pseudomonas Syringae Infection Assay

The infection assay was conducted according to [43], using the strain *P. syringae* pv *tomato* DC3000. Arabidopsis plants were grown on half strength MS medium with 0.3% phytagel in a growth chamber at 24 °C with a 12 h light/12 h dark photoperiod for 2 weeks. *P. syringae* was cultured on King’s B medium, and for inoculation, the cells were suspended in 10 mM MgCl_2_ at a concentration of 5 × 10^5^ CFU/mL. Arabidopsis seedlings were inoculated by flooding the plate with the *P. syringae* suspension until the seedlings were completely submerged in the inoculum. The *P. syringae* suspension was removed from the plates and the plates with the inoculated seedlings were put back into the growth chamber. One hour after the inoculation and at 3 dpi, 4 seedlings (only aerial parts) of each line were removed and surface sterilized with 5% H_2_O_2_ for 3 min and washed three times with sterile distilled water. The pooled sample of 4 seedlings was homogenized in 10 mL sterile distilled water using a mortar and pestle. Diluted samples were plated onto King’s B medium containing rifampicin (50 μg/mL) and colonies were counted after 24 h using proper diluted samples. Data were normalized as CFU/mg using the total weight of inoculated seedlings. Bacterial numbers were evaluated in three independent experiments.

### 4.6. Resistance Test against Botrytis Cinerea

Arabidopsis plants were grown on soil under short day conditions (8 h light, 16 h dark). *B. cinerea* was grown on potato dextrose media (38 g/liter potato dextrose agar). After two weeks, the spores of the fungus were harvested in sterile water and passed through sterile cotton cloth. Spores were counted with a hemocytometer (Thoma, area 0.0025 mm^2^, depth 0.1 mm, LO-Laboroptik, Lancing, UK). The spore suspension was adjusted to 10^5^ spores/mL using ½ strength potato dextrose broth. Drops of 5 μL spore suspension were deposited on the upper surfaces of 4-week-old plants on the left or right side to ensure uniformity across all leaves in the visual observations. The plants were covered with plastic coverings to maintain high humidity. The lesion diameter was determined with the help of a meter rod at 5 dpi and a photo of each leaf was taken with a camera (Digital Camera Nikon Coolpix S8200, Nikon, Japan). For each line, 3 plants were used and the experiment was performed in triplicate.

### 4.7. Semi-Quantitative RT-PCR of Overexpression Lines

Total RNA was purified from seedlings using the “NucleoSpin^®^ RNA Plant” from Macherey-Nagel (Fisher Scientific, Vienna, Austria). A volume of 2 μL of eluted RNA was used for photometric measurement of RNA using NanoDrop (NanoDrop™ 2000c, PEQLAB). Semi-quantitative RT-PCR was conducted using the “One-step Master Mix RT-PCR Kit” (Affymetrix, Santa Clara, California, USA) according to the manufacturer’s instructions (Figure S9). Primers are listed in Table S4.

### 4.8. GUS Reporter Analysis

Histochemical detection of GUS activity was performed according to [44], as described by [45], using X-gluc (Biomol, Hamburg, Germany) in 0.1 M sodium phosphate buffer pH 7.0, 0.1% Triton-X 100, 0.5 mM K_3_[Fe(CN)_6_], 0.5 mM K_4_[Fe(CN)_6_] and 10 mM Na_2_EDTA. Plant parts were incubated in X-gluc solution at 37 °C. After staining, chlorophyll was removed from photosynthetic tissues with 70% (*v*/*v*) ethanol. The seedlings and different plant parts were stained before and after flowering. Similarly, for GUS staining of syncytia and galls, the infected roots of promoter::GUS plants were incubated with X-gluc at 37 °C. The staining in syncytia, galls and uninfected roots was performed at different time points and photographed under an inverse microscope (Axiovert 200M; Zeiss, Hallerbergmoos, Germany) with an integrated camera (AxioCam MRc5; Zeiss, Hallerbergmoos, Germany).

### 4.9. Sequence Retrieval and Characterization of Rboh Family Genes

Genomic DNA, coding (CDs) and protein sequences of all *Rboh* genes of *Arabidopsis thaliana* were retrieved from the Arabidopsis Information Resource (TAIR) website (https://www. arabidopsis.org/) in FASTA format. All other characters of *Rboh* genes mentioned in Table S2, such as chromosomal number, strand type, number of exons, length of protein, coding (CDs) and genomic DNA (gDNA), gene location on chromosome and isoelectric point (pI) of protein, were also taken from the TAIR website. Grand average of hydropathy (GRAVY) of *Rboh* proteins was calculated using the Sequence Manipulation Suite online website (https://www.bioinformatics.org/sms 2/protein gravy.html). Signal peptide cleavage site (SPCS) was predicted through SignalP 4.1 Server (http://www.cbs.dtu.dk/services/SignalP-4.1/).

### 4.10. Chromosomal Mapping and Gene Structure Analyses of Rboh Genes

Chromosomal positioning of 10 Arabidopsis *Rboh* genes was performed to display their genome-wide distribution. For positioning of the genes on their respective chromosomes, MapChart [46] desktop-based software was used with default parameters. Gene structure analysis was performed to analyze the intron-exon pattern of 10 *Rboh* genes. For this purpose, online Gene Structure Display Server 2.0 (http://gsds.gao-lab.org/) (Center for Bioinformatics (CBI), Peking University, Beijing, China) was used [47]. Genomic DNA and coding (CDs) sequences of all genes were arranged according to the groups formed by the phylogenetic analysis. Thereafter, arranged gDNA and CD sequences were used to map the untranslated regions, number of exons and number of introns in all sequences.

### 4.11. Phylogenetic Analysis

An evolutionary tree of 10 *Rboh* protein sequences was constructed using Molecular Evolutionary Genetic Analysis software (MEGA version 7.0, Penn State University, State College, Pennsylvania, USA) [48]. First, all protein sequences were subjected to alignment through the MUSCLE algorithm with default parameters such as gap opening penalty -2.9, gap extension penalty 0 and hydrophobicity multiplier 1.2, and the UPGMBA (Unweighted Pair Group Method with arithmetic mean) clustering method was used. Thereafter, using aligned data, the evolutionary history was inferred using the maximum likelihood method based on the JTT (Jones-Taylor-Thornton) matrix-based model (MEGA version 7.0, Penn State University, State College, Pennsylvania, USA) [49]. Furthermore, initial tree(s) for the heuristic search were obtained automatically by applying Neighbor-Join and BioNJ algorithms to a matrix of pairwise distances estimated using a JTT model and then selecting the topology with superior log likelihood value. From a total of 780 positions, the tree with the highest log likelihood value of -11668.24 was developed using 1000 bootstrap replications, partial gap deletion and 95% site coverage cutoff value.

### 4.12. Conserved Motif and Domain Analysis

To discover conserved motifs in Rboh protein sequences, conserved motif analysis was performed using the online (http://meme-suite.org/) MEME SUITE tool [50]. To display the conserved domains through identified motifs, different parameters were assessed one by one for motif discovery; however, the maximum number of motifs of 15, minimum motif width of 20 and maximum motif width of 220 were finally used. All protein sequences of *Rboh* genes were arranged according to their clustering in the phylogenetic tree. Conserved domain analysis was performed using the online InterPro database (https://www.ebi.ac.uk/interpro/) with default parameters.

### 4.13. Statistical Analysis

Data regarding the number of nematodes, number of galls, size of female nematodes and syncytia, number of CFU and lesion diameter were analyzed using single factor ANOVA (*p* < 0.05), while the means comparisons were performed using the least significant difference (LSD) test at a 95% level of confidence.

## 5. Conclusions

We have shown that *RbohB* is a root-specific gene which is strongly downregulated in syncytia induced by *H. schachtii* in Arabidopsis roots. Overexpression resulted in resistance against different pathogens. In contrast, expression of *RbohD*/*RbohF* in syncytia is needed for full susceptibility to *H. schachtii*. Since the Rboh proteins are very similar, the different behavior of *RbohB* and *RbohD*/*RbohF* might be due to different interaction partners.

## Supplementary Materials

Supplementary materials can be found at https://www.mdpi.com/1422-0067/21/15/5556/s1. Table S1. Expression of Rboh genes in syncytia. Table S2. Characteristics of Arabidopsis Rboh family genes and proteins. Table S3. Description of the motifs identified in Arabidopsis Rboh proteins. Table S4. Primers used in this work (restriction sites are underlined). Figure S1. Leaves of the RbohB promoter::GUS line were stained for GUS at different time points after infection with A, A. brassicicola; B, B. cinerea; C, P. syringae. Figure S2. Sequence logos of the predicted 15 motifs in Rboh protein sequences. Figure S3. Description of predicted domains in Arabidopsis Rboh proteins. Figure S4. Conserved domains predicted in the Arabidopsis Rboh proteins. Figure S5. Alignment of RbohB, RbohD and RbohF protein sequences. Figure S6. Expression of Arabidopsis Rboh genes in anatomical parts according to Genevestigator. Figure S7. Expression of Arabidopsis Rboh genes after infection with pathogens according to Genevestigator. Figure S8. Expression of Arabidopsis Rboh genes after treatment with elicitors according to Genevestigator. Figure S9. RT-PCR of overexpression lines of RbohB.

## Abbreviations

Dpi	Days post inoculation;
Rboh	Respiratory burst oxidase homologue;
ROS	Reactive oxygen species;
CFU	Colony forming units
