# Supplementary material for: The Arabidopsis RboHB Encoded by At1g09090 Is Important for Resistance against Nematodes
Source: Int J Mol Sci. 2020 Aug 3;21(15):5556. doi: 10.3390/ijms21155556 (PMC7432757; doi:10.3390/ijms21155556)
Supplement: Supplementary file 1 [file ijms-21-05556-s001.pdf]

International Journal of
Molecular Sciences

MDPI

Supplementary materials

Table S1. Expression of *Rboh* genes in syncytia.

		Syncytium	Root
At5g07390	*RbohA*	3.9	6.6
At1g09090	*RbohB*	1.9	12.0
At5g51060	*RbohC*	5.7	8.8
At5g47910	*RbohD*	8.0	8.4
At1g19230	*RbohE*	2.9	4.6
At1g64060	*RbohF*	5.5	7.3
At4g25090	*RbohG*	3.3	4.4
At5g60010	*RbohH*	2.7	2.8
At4g11230	*RbohI*	2.3	3.1
At3g45810	*RbohJ*	2.7	3.0

Expression data from Szakasits et al. (The transcriptome of syncytia induced by the cyst nematode Heterodera schachtii in Arabidopsis roots (2009) Plant Journal, **57**, 771–784) from a GeneChip analysis of *H. schachtii* syncytia in Arabidopsis roots showing normalized data on a log2 scale. Significant downregulation (as from the original data analysis) in syncytia compared to control roots is marked blue.

Table S2. Characteristics of Arabidopsis *Rboh* family genes and proteins.

Gene name	Accession Number	Chrom0s0me #	Exons	Gene position on chromosome	Orientation	gDNA (bp)	CD (bp)	Protein (aa)	MW (Da)	pI	GRAVY	SPCS
AtRbohA	At5g07390	5	12	2335895-2339913	Reverse	4019	2709	902	102934.6	9.54	-0.229	No
AtRbohB	At1g09090	1	12	2932739-2936586	Forward	3848	2532	843	96389.3	9.59	-0.156	No
AtRbohC	At5g51060	5	11	20757284-20762431	Reverse	5148	2718	905	102517.4	9.91	-0.211	No
AtRbohD	At5g47910	5	8	19397443-19402061	Forward	4619	2766	921	103907.7	9.68	-0.241	No
AtRbohE	At1g19230	1	14	6643942-6649149	Forward	5208	2859	952	107701.6	8.9	-0.207	No
AtRbohF	At1g64060	1	14	23769774-23776984	Forward	7211	2835	944	108417.3	9.54	-0.287	No
AtRbohG	At4g25090	4	11	12878667-12883805	Reverse	5139	2550	849	96861.7	9.41	-0.196	No
AtRbohH	At5g60010	5	11	24160270-24165052	Forward	4783	2661	886	100626.7	9.5627	-0.196	No

AtRbohI	At4g11230	4	10	6840473-6845627	Reverse	5155	2826	941	106951	8.58	-0.230	No
AtRbohJ	At3g45810	3	13	16832726-16837792	Reverse	5067	2739	912	102936.2	9.9	-0.203	No

gDNA = genomic DNA, CD = coding sequence, MW = molecular weight, pI = isoelectric point, GRAVY = grand average of hydropathy, SPCS = signal peptide cleavage site.

Table S3. Description of the motifs identified in Arabidopsis Rboh proteins.

Motif	E-value	Sites	Width	Consensus sequence
1	3.5^{e-773}	10	136	KRAYFYWVTREQGSFDWFKGVMBEIAEYDKKGVIELHNYLTSVYEEGDARSALITMLQSLNHAKNGVDIVSGTRVRTHFARPNWRKV FKKIAVKHPNARIGVFYCGAPTLVKELKKLAQDFSHKTSTRFEFHKENF
2	3.0^{e-470}	10	93	RAFRSSIKAVKILKVAVYPGNVLSLHMSKPTGFKYKSGQYMFVNCPAVSPFEWHPFSITSAPGDDYLSVHIRALGDWTEELRSVFSEVCK PPP
3	6.3^{e-382}	10	77	YFLLDNWKRIWVLALWIIIMAILFTWKFIZYKRKPAYEVMGYCVCVAKGAAETLKLNMALILLPVCRNTITWLRSKT
4	3.2^{e-382}	9	90	ITKEZLKEFWEQITDKSFDSRLQIFFDMVDKBEDGRJTEEEVKEIIVLSASANKLSNJKKQADEYAALIMEELDPDNJGYIELEQLETLL
5	6.5^{e-236}	10	39	DGPYGAPAQDYKKYDVLLLVGLGIGATPFISILKDJLNN
6	3.2^{e-184}	20	28	PFDDNIWFHKTIWMYJAVPVLLYAGERL
7	9.3^{e-145}	10	33	LPKPLKKLTGFNAFWYSHHLFVIVYILLIVHGY
8	2.1^{e-115}	10	38	QPPTYLGLVKGPEGITGILMVILMLIAFTLATTWFRRN
9	6.2^{e-072}	10	38	TGGLPKRKFGGCIGMMDSKEFALELFDALARRRRIKGE
10	1.1^{e-037}	10	26	ARLDRTKSSAERALKGLKFISKTDGG
11	8.4^{e-036}	10	20	CDFPRLJAATEDEYEPYAKY
12	$4.^{2e-015}$	7	28	SGEKKKLSQMLSQKLIPTRDRNPLKRWY
13	1.6^{e-007}	6	23	SRNRDEEYVEITLDIQDDSVSVH
14	8.4^{e-005}	2	28	PKPNDLIRMETRARGVNPHIEESQVLFP
15	2.3^{e-004}	2	41	KNVSKNLGVGSIIRSLSVNKWRKSGNLGSPSTRKSGNLGPP

Table S4. Primers used in this work (restriction sites are underlined).

Name	Sequence	Amplification
At1g09090forNco	TAGTCCATGGGGAGGAAGAAATGG	Protein coding sequence of *At1g09090* from cDNA
At1g09090revBam	TGATGGATCCATAGAATCGAAACTA	
At1g09090Mrev	ATAAGAACCACCATTGTAACACCGGT	Eliminate internal NcoI site
At1g09090Mfor	ACCGGTGTTACAATGGTGGTTCTTAT	
pRBOHBforKpn	GTTGGTACCTCTTCTTCGATCTCG	Promoter fragment of *At1g09090*
pRBOHBrevNco	CCTCCCCCATGGACTACAATTAC	
RbohRTfor	ACCGGTGTTACAATGGTGGTTCTTAT	RT-PCR of Rboh overexpression lines
RbohRTrev	TTGAGTTGCGATGTCCAGTC	

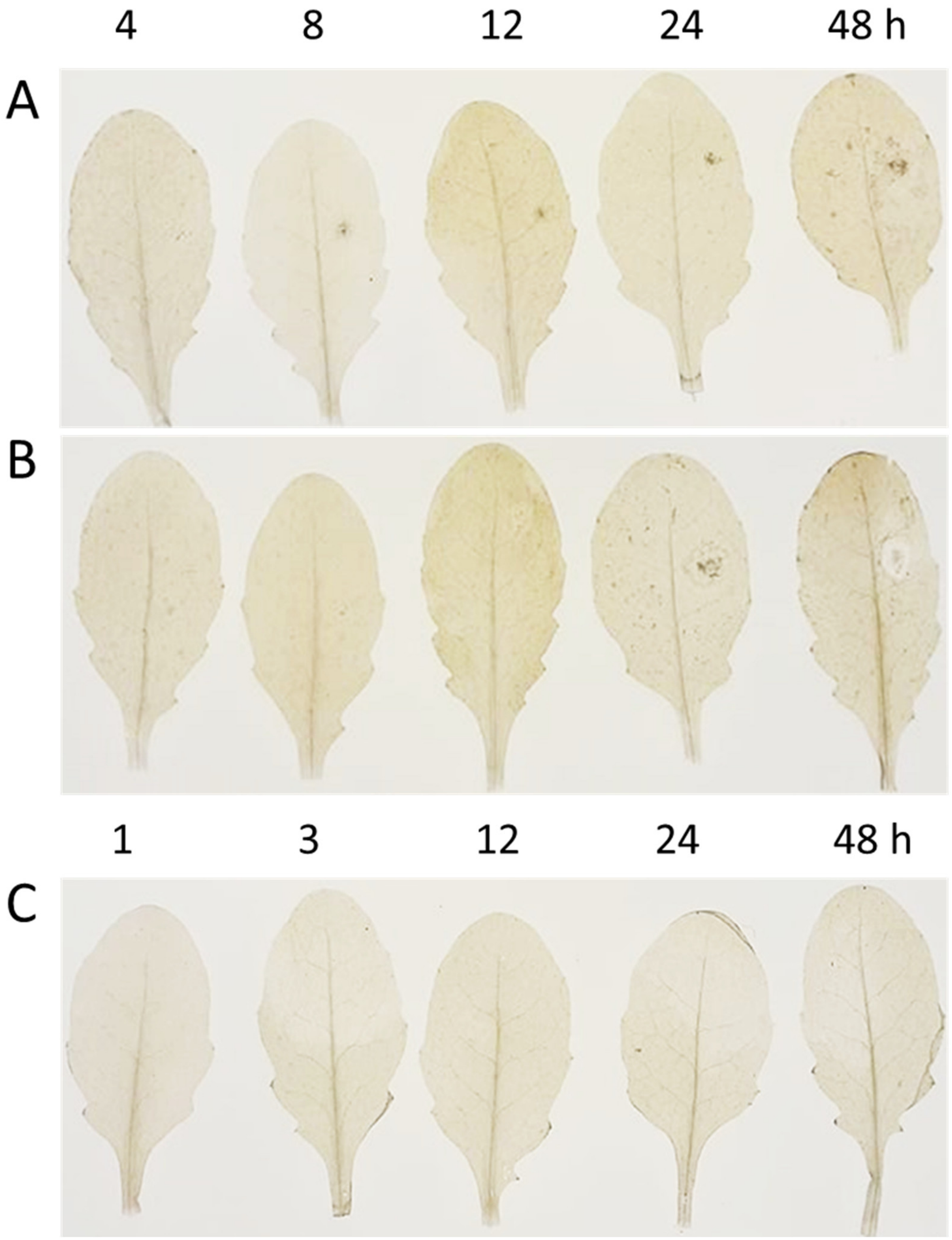

Figure S1. Leaves of the *RbohB* promoter::GUS line were stained for GUS at different time points after infection with A, *A. brassicicola*; B, *B. cinerea*; C, *P. syringae*.

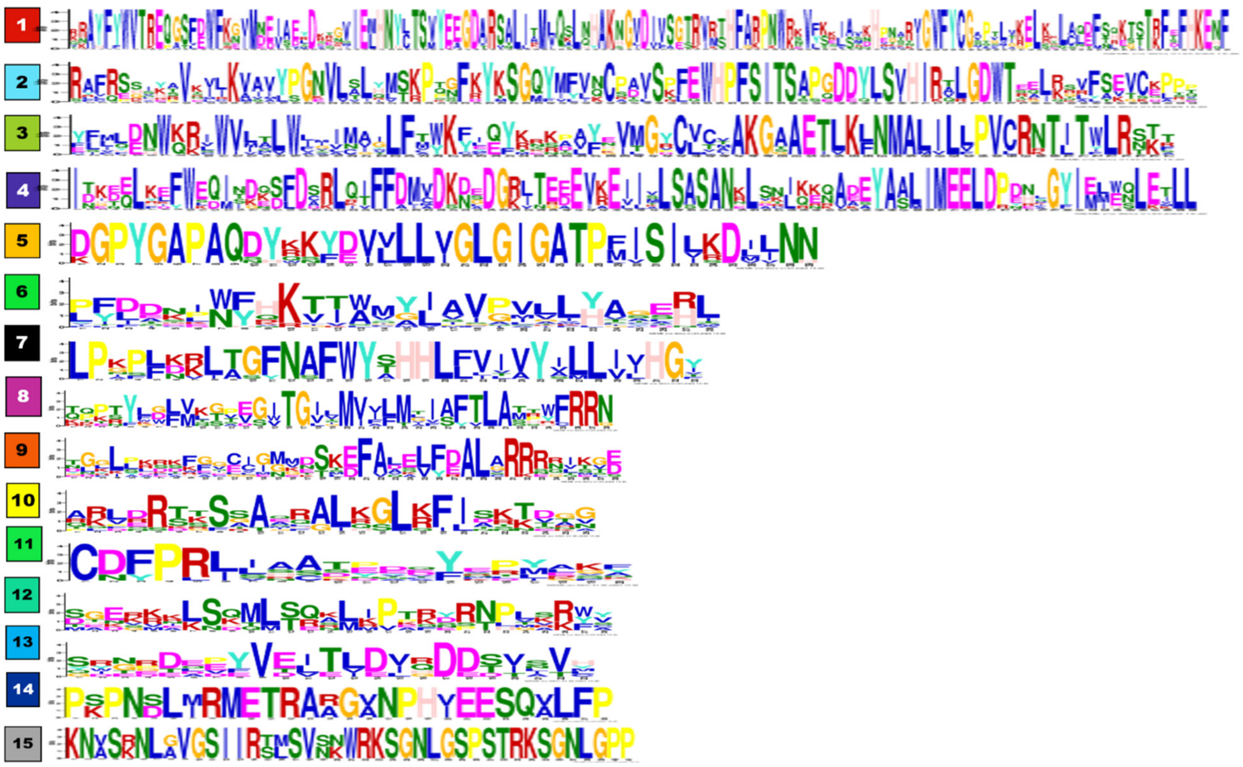

Figure S2. Sequence logos of the predicted 15 motifs in Rboh protein sequences. Colors of the motifs are the same as in Figure 11. Each letter shows one letter amino acid code. Amino acids with higher letters are more conserved.

AtRbohG

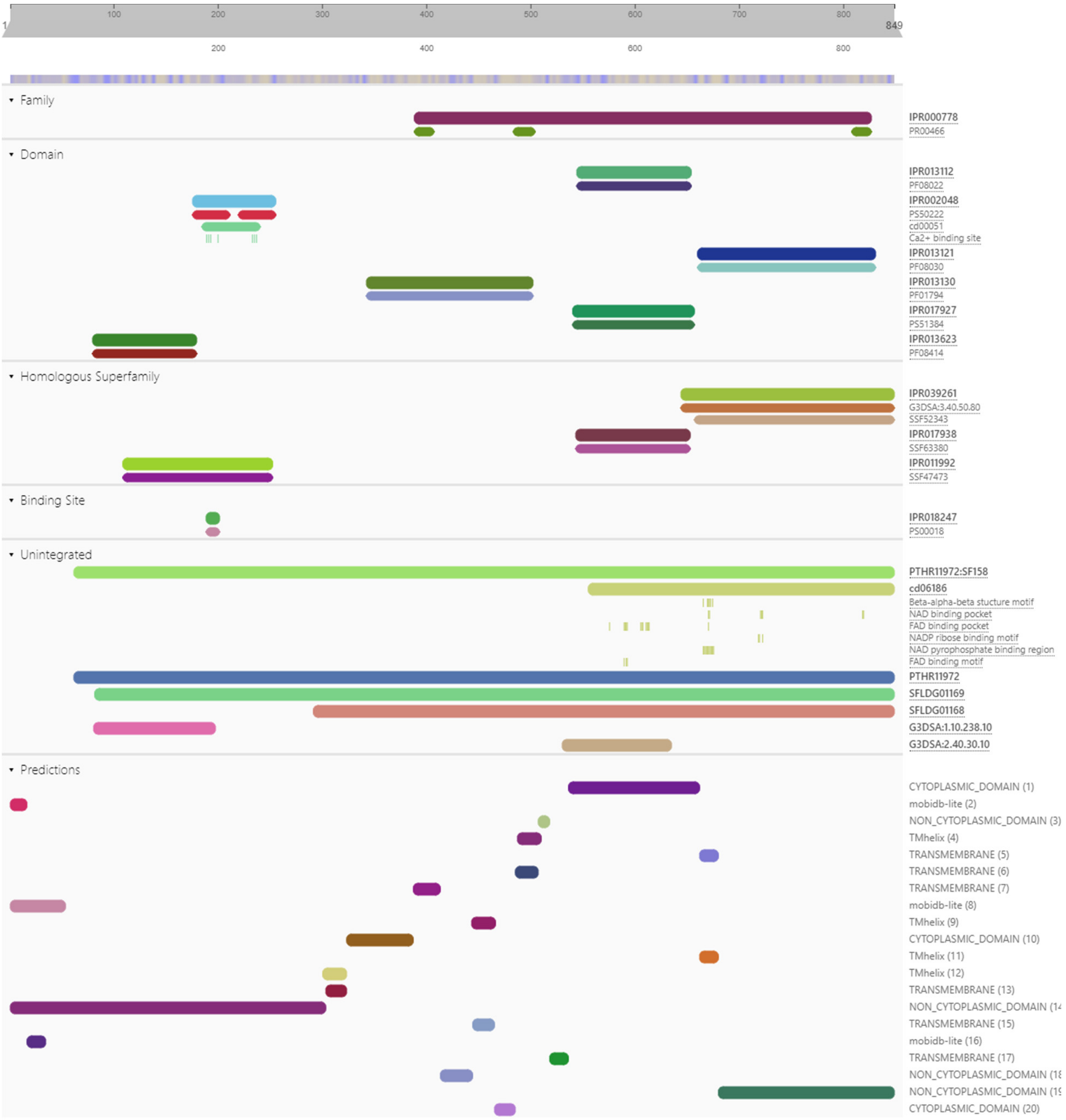

1
100
200
300
400
500
600
700
800
849
200
400
600
800
Family
IPR000778
PR00466
Domain
IPR013112
PF08022
IPR002048
PS50222
cd00051
Ca2+ binding site
IPR013121
PF08030
IPR013130
PF01794
IPR017927
PS51384
IPR013623
PF08414
Homologous Superfamily
IPR039261
G3DSA:3.40.50.80
SSF52343
IPR017938
SSF63380
IPR011992
SSF47473
Binding Site
IPR018247
PS00018
Unintegrated
PTHR11972:SF158
cd06186
Beta-alpha-beta stucture motif
NAD binding pocket
FAD binding pocket
NADP ribose binding motif
NAD pyrophosphate binding region
FAD binding motif
PTHR11972
SFLDG01169
SFLDG01168
G3DSA:1.10.238.10
G3DSA:2.40.30.10
Predictions
CYTOPLASMIC_DOMAIN (1)
mobidb-lite (2)
NON_CYTOPLASMIC_DOMAIN (3)
TMhelix (4)
TRANSMEMBRANE (5)
TRANSMEMBRANE (6)
TRANSMEMBRANE (7)
mobidb-lite (8)
TMhelix (9)
CYTOPLASMIC_DOMAIN (10)
TMhelix (11)
TMhelix (12)
TRANSMEMBRANE (13)
TRANSMEMBRANE (15)
mobidb-lite (16)
TRANSMEMBRANE (17)
CYTOPLASMIC_DOMAIN (20)

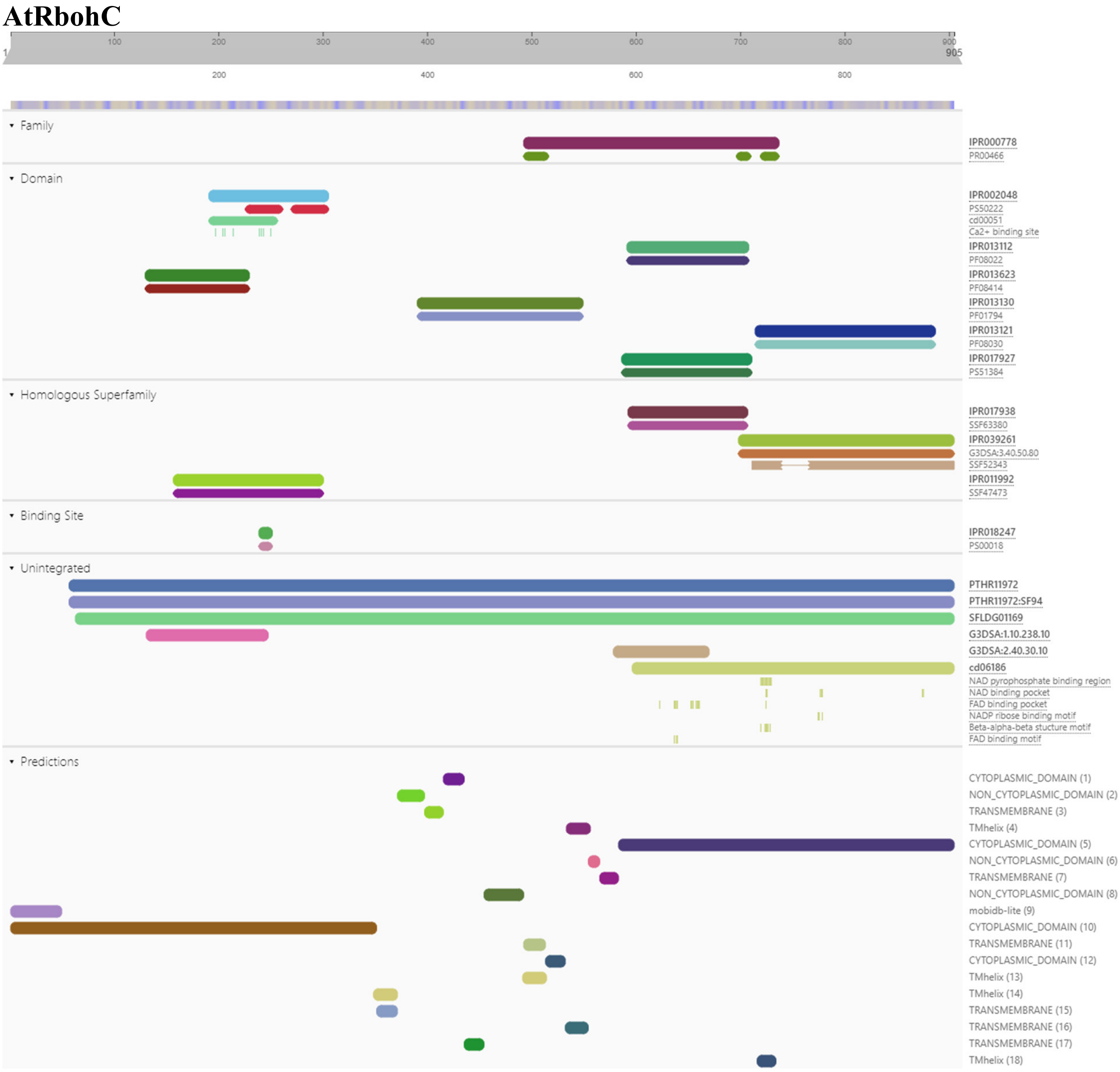
AtRbohC
Family
IPR000778
PR00466
Domain
IPR002048
PS50222
cd00051
Ca2+ binding site
IPR013112
PF08022
IPR013623
PF08414
IPR013130
PF01794
IPR013121
PF08030
IPR017927
PS51384
Homologous Superfamily
IPR017938
SSF63380
IPR039261
G3DSA:3.40.50.80
SSF52343
IPR011992
SSF47473
Binding Site
IPR018247
PS00018
Unintegrated
PTHR11972
PTHR11972:SF94
SFLDG01169
G3DSA:1.10.238.10
G3DSA:2.40.30.10
cd06186
NAD pyrophosphate binding region
NAD binding pocket
FAD binding pocket
NADP ribose binding motif
Beta-alpha-beta stucture motif
FAD binding motif
Predictions
CYTOPLASMIC_DOMAIN (1)
NON_CYTOPLASMIC_DOMAIN (2)
TRANSMEMBRANE (3)
TMhelix (4)
CYTOPLASMIC_DOMAIN (5)
NON_CYTOPLASMIC_DOMAIN (6)
TRANSMEMBRANE (7)
NON_CYTOPLASMIC_DOMAIN (8)
mobidb-lite (9)
CYTOPLASMIC_DOMAIN (10)
TRANSMEMBRANE (11)
CYTOPLASMIC_DOMAIN (12)
TMhelix (13)
TMhelix (14)
TRANSMEMBRANE (15)
TRANSMEMBRANE (16)
TRANSMEMBRANE (17)
TMhelix (18)

AtRbohA

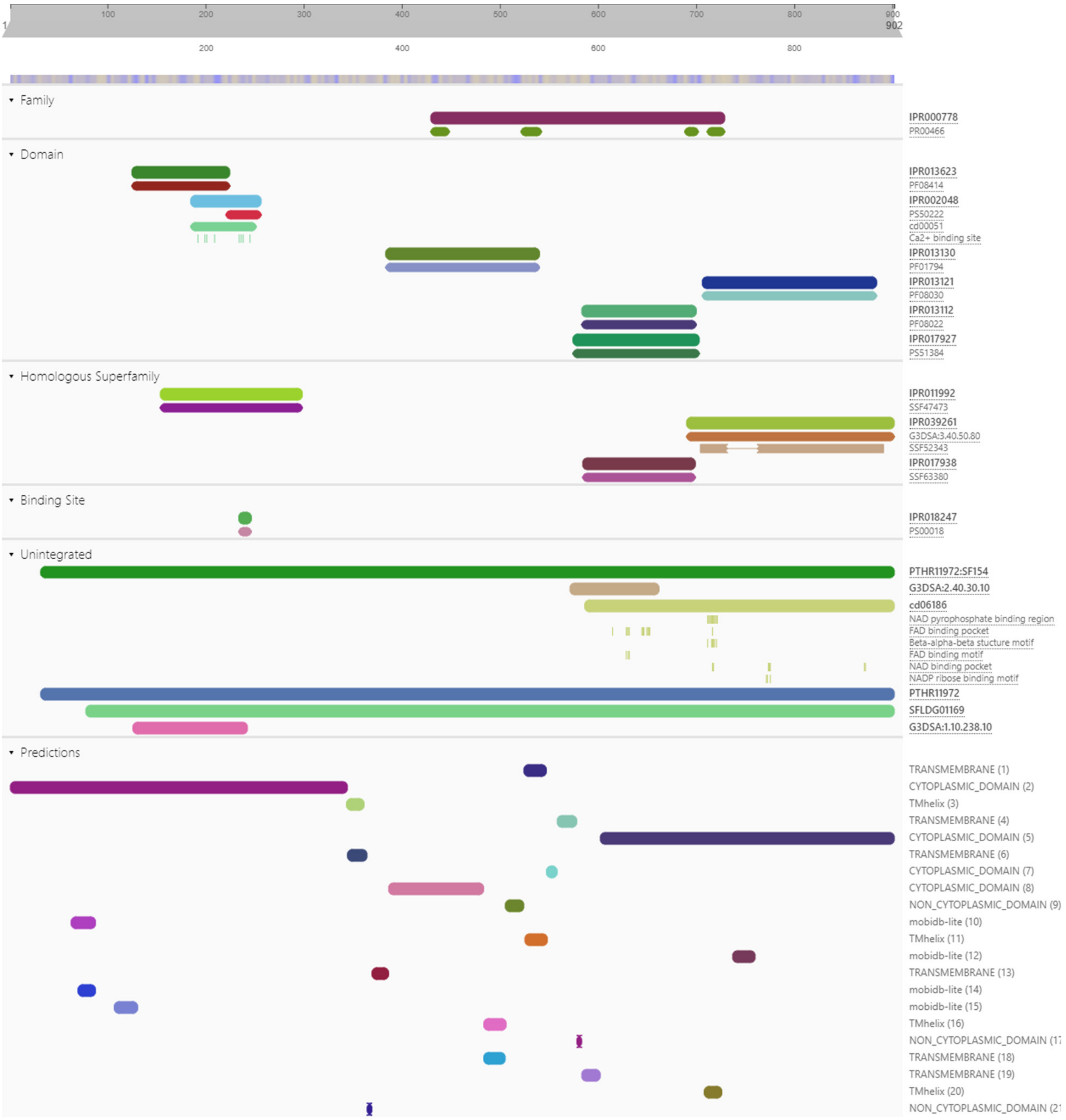
1
100
200
300
400
500
600
700
800
900
902
Family
IPR000778
PR00466
Domain
IPR013623
PF08414
IPR002048
PS50222
cd00051
Ca2+ binding site
IPR013130
PF01794
IPR013121
PF08030
IPR013112
PF08022
IPR017927
PS51384
Homologous Superfamily
IPR011992
SSF47473
IPR039261
G3DSA:3.40.50.80
SSF52343
IPR017938
SSF63380
Binding Site
IPR018247
PS00018
Unintegrated
PTHR11972:SF154
G3DSA:2.40.30.10
cd06186
NAD pyrophosphate binding region
FAD binding pocket
Beta-alpha-beta stucture motif
FAD binding motif
NAD binding pocket
NADP ribose binding motif
PTHR11972
SFLDG01169
G3DSA:1.10.238.10
Predictions
TRANSMEMBRANE (1)
CYTOPLASMIC_DOMAIN (2)
TMhelix (3)
TRANSMEMBRANE (4)
CYTOPLASMIC_DOMAIN (5)
TRANSMEMBRANE (6)
CYTOPLASMIC_DOMAIN (7)
CYTOPLASMIC_DOMAIN (8)
NON_CYTOPLASMIC_DOMAIN (9)
mobidb-lite (10)
TMhelix (11)
mobidb-lite (12)
TRANSMEMBRANE (13)
mobidb-lite (14)
mobidb-lite (15)
TMhelix (16)
TRANSMEMBRANE (18)
TRANSMEMBRANE (19)
TMhelix (20)

AtRbohD

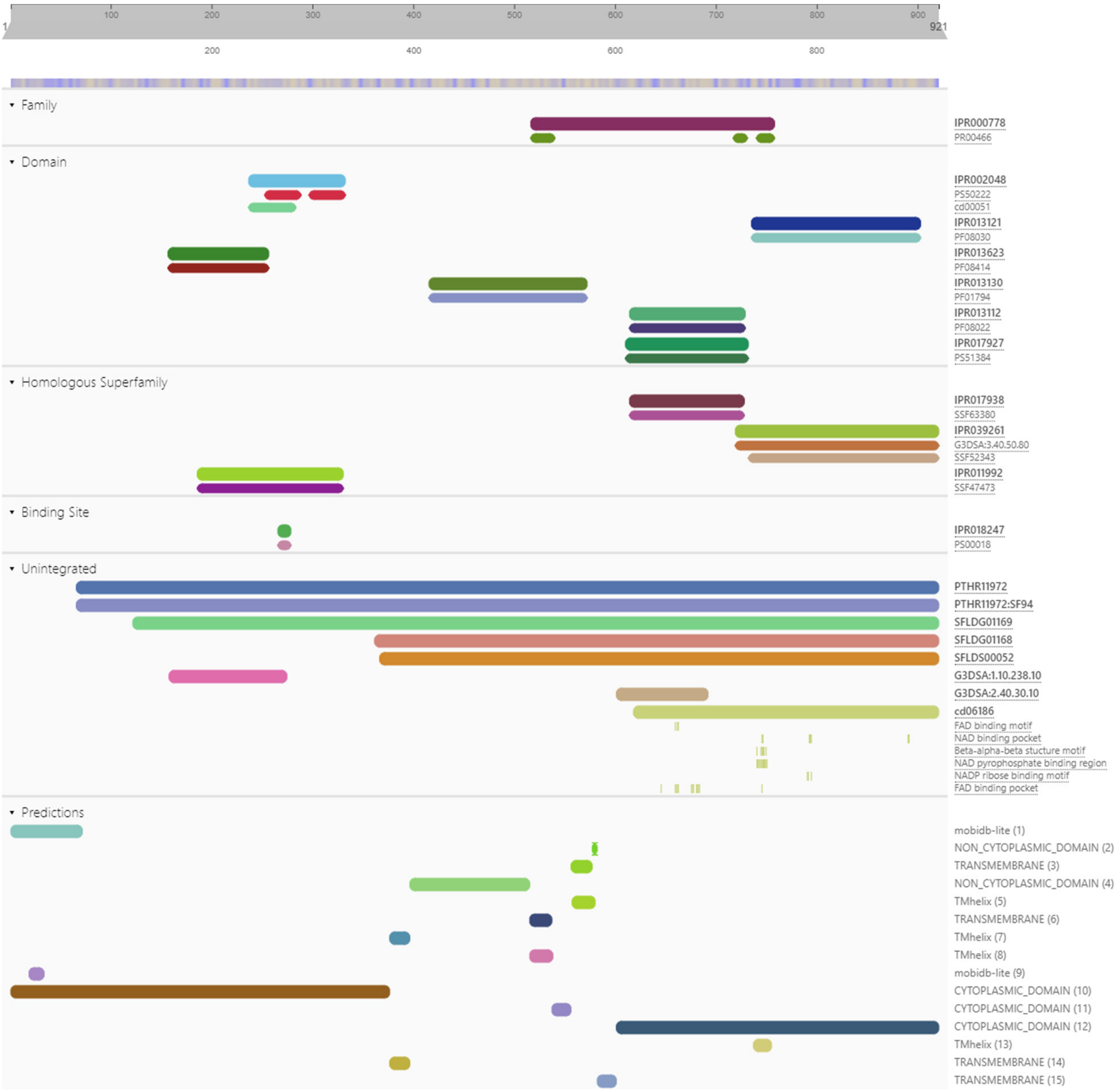
1
921
Family
IPR000778
PR00466
Domain
IPR002048
PS50222
cd00051
IPR013121
PF08030
IPR013623
PF08414
IPR013130
PF01794
IPR013112
PF08022
IPR017927
PS51384
Homologous Superfamily
IPR017938
SSF63380
IPR039261
G3DSA:3.40.50.80
SSF52343
IPR011992
SSF47473
Binding Site
IPR018247
PS00018
Unintegrated
PTHR11972
PTHR11972:SF94
SFLDG01169
SFLDG01168
SFLDS00052
G3DSA:1.10.238.10
G3DSA:2.40.30.10
cd06186
FAD binding motif
NAD binding pocket
Beta-alpha-beta stucture motif
NAD pyrophosphate binding region
NADP ribose binding motif
FAD binding pocket
Predictions
mobidb-lite (1)
NON_CYTOPLASMIC_DOMAIN (2)
TRANSMEMBRANE (3)
NON_CYTOPLASMIC_DOMAIN (4)
TMhelix (5)
TRANSMEMBRANE (6)
TMhelix (7)
TMhelix (8)
mobidb-lite (9)
CYTOPLASMIC_DOMAIN (10)
CYTOPLASMIC_DOMAIN (11)
CYTOPLASMIC_DOMAIN (12)
TMhelix (13)
TRANSMEMBRANE (14)
TRANSMEMBRANE (15)

AtRbohB

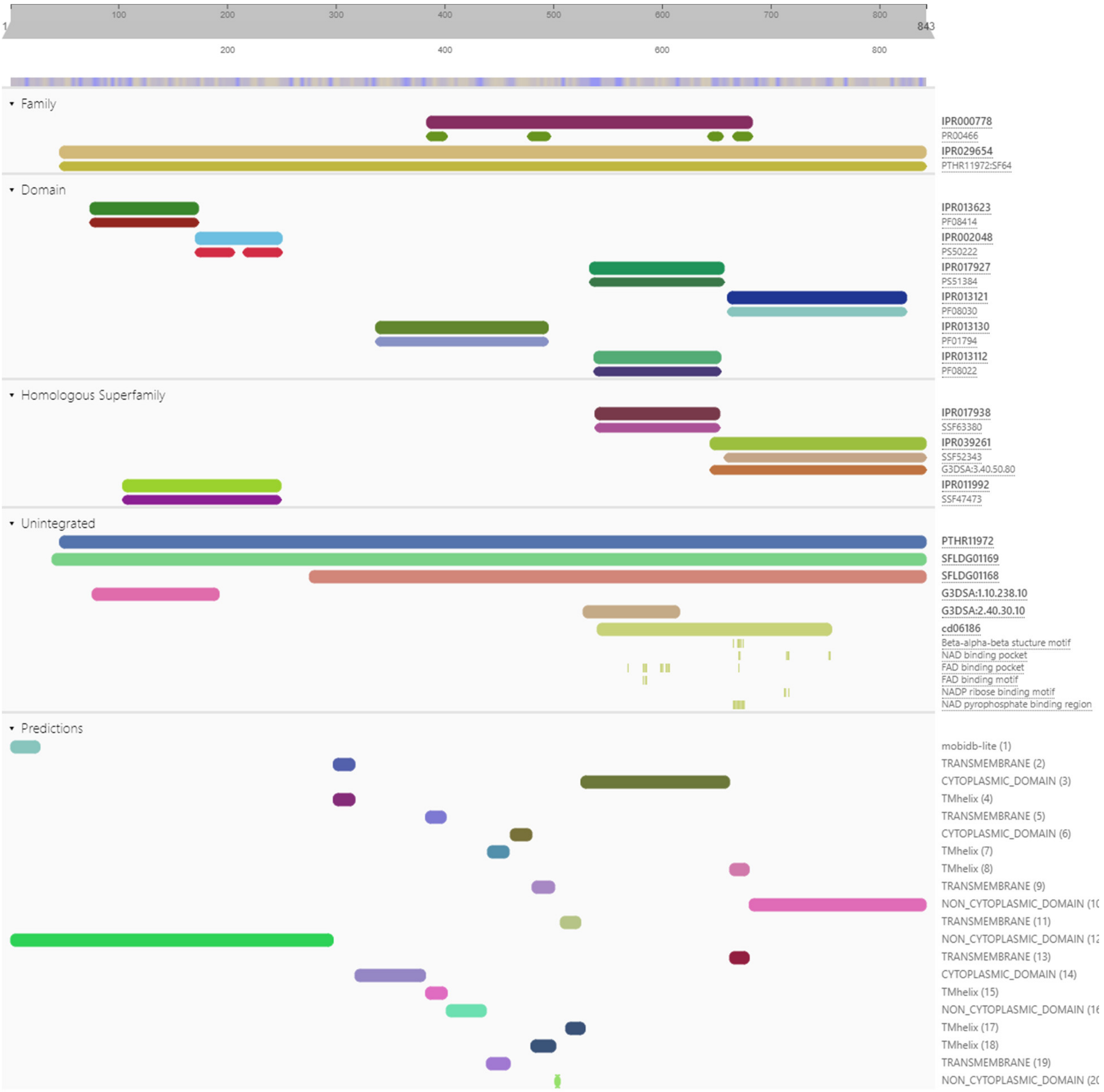
Family
IPR000778
PR00466
IPR029654
PTHR11972:SF64
Domain
IPR013623
PF08414
IPR002048
PS50222
IPR017927
PS51384
IPR013121
PF08030
IPR013130
PF01794
IPR013112
PF08022
Homologous Superfamily
IPR017938
SSF63380
IPR039261
SSF52343
G3DSA:3.40.50.80
IPR011992
SSF47473
Unintegrated
PTHR11972
SFLDG01169
SFLDG01168
G3DSA:1.10.238.10
G3DSA:2.40.30.10
cd06186
Beta-alpha-beta stucture motif
NAD binding pocket
FAD binding pocket
FAD binding motif
NADP ribose binding motif
NAD pyrophosphate binding region
Predictions
mobidb-lite (1)
TRANSMEMBRANE (2)
CYTOPLASMIC_DOMAIN (3)
TMhelix (4)
TRANSMEMBRANE (5)
CYTOPLASMIC_DOMAIN (6)
TMhelix (7)
TMhelix (8)
TRANSMEMBRANE (9)
TRANSMEMBRANE (11)
TRANSMEMBRANE (13)
CYTOPLASMIC_DOMAIN (14)
TMhelix (15)
TMhelix (17)
TMhelix (18)
TRANSMEMBRANE (19)

AtRbohJ

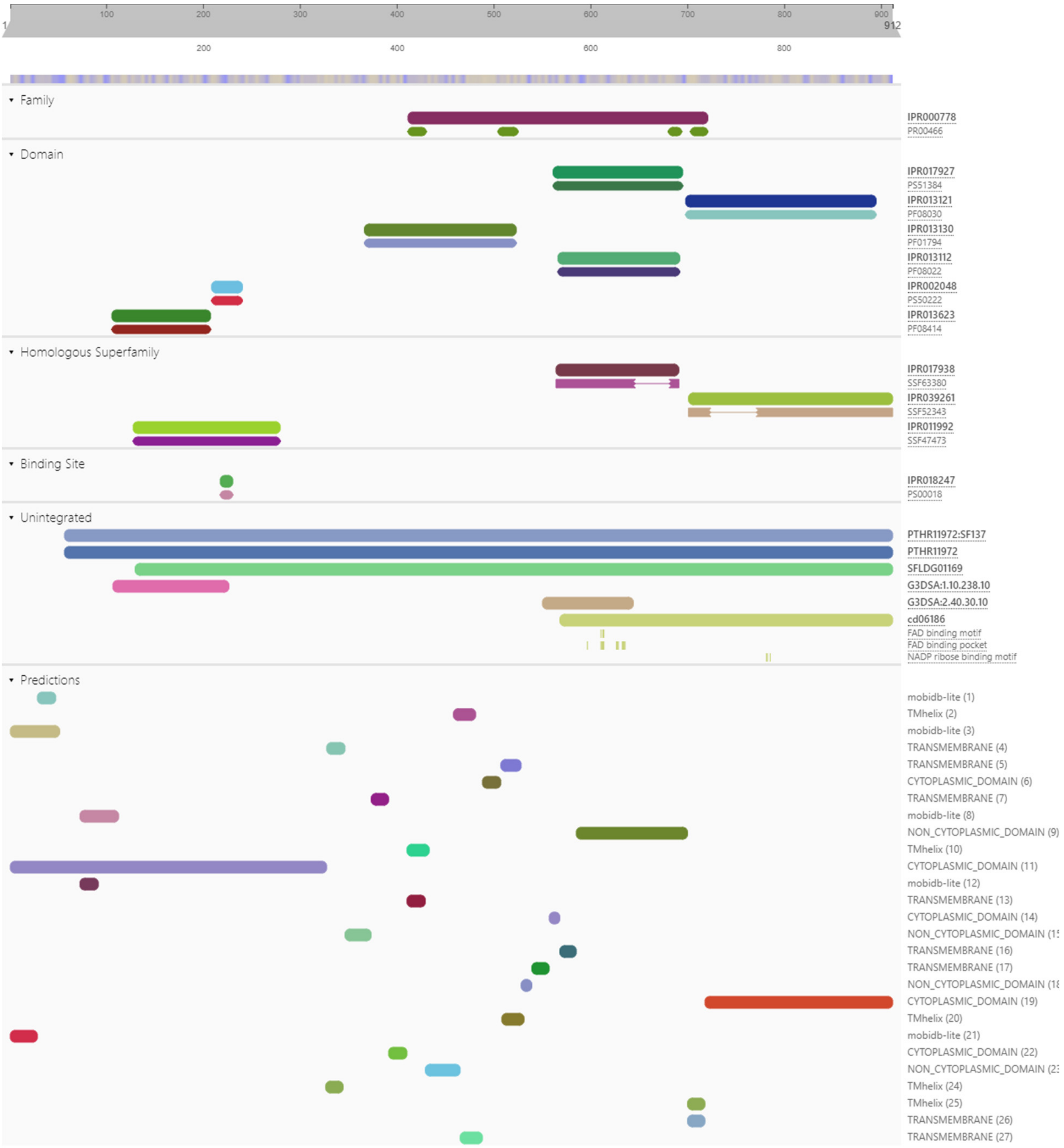
Family
IPR000778
PR00466
Domain
IPR017927
PS51384
IPR013121
PF08030
IPR013130
PF01794
IPR013112
PF08022
IPR002048
PS50222
IPR013623
PF08414
Homologous Superfamily
IPR017938
SSF63380
IPR039261
SSF52343
IPR011992
SSF47473
Binding Site
IPR018247
PS00018
Unintegrated
PTHR11972:SF137
PTHR11972
SFLDG01169
G3DSA:1.10.238.10
G3DSA:2.40.30.10
cd06186
FAD binding motif
FAD binding pocket
NADP ribose binding motif
Predictions
mobidb-lite (1)
TMhelix (2)
mobidb-lite (3)
TRANSMEMBRANE (4)
TRANSMEMBRANE (5)
CYTOPLASMIC_DOMAIN (6)
TRANSMEMBRANE (7)
mobidb-lite (8)
NON_CYTOPLASMIC_DOMAIN (9)
TMhelix (10)
CYTOPLASMIC_DOMAIN (11)
mobidb-lite (12)
TRANSMEMBRANE (13)
CYTOPLASMIC_DOMAIN (14)
TRANSMEMBRANE (16)
TRANSMEMBRANE (17)
CYTOPLASMIC_DOMAIN (19)
TMhelix (20)
mobidb-lite (21)
CYTOPLASMIC_DOMAIN (22)
TMhelix (24)
TMhelix (25)
TRANSMEMBRANE (26)
TRANSMEMBRANE (27)

AtRbohH

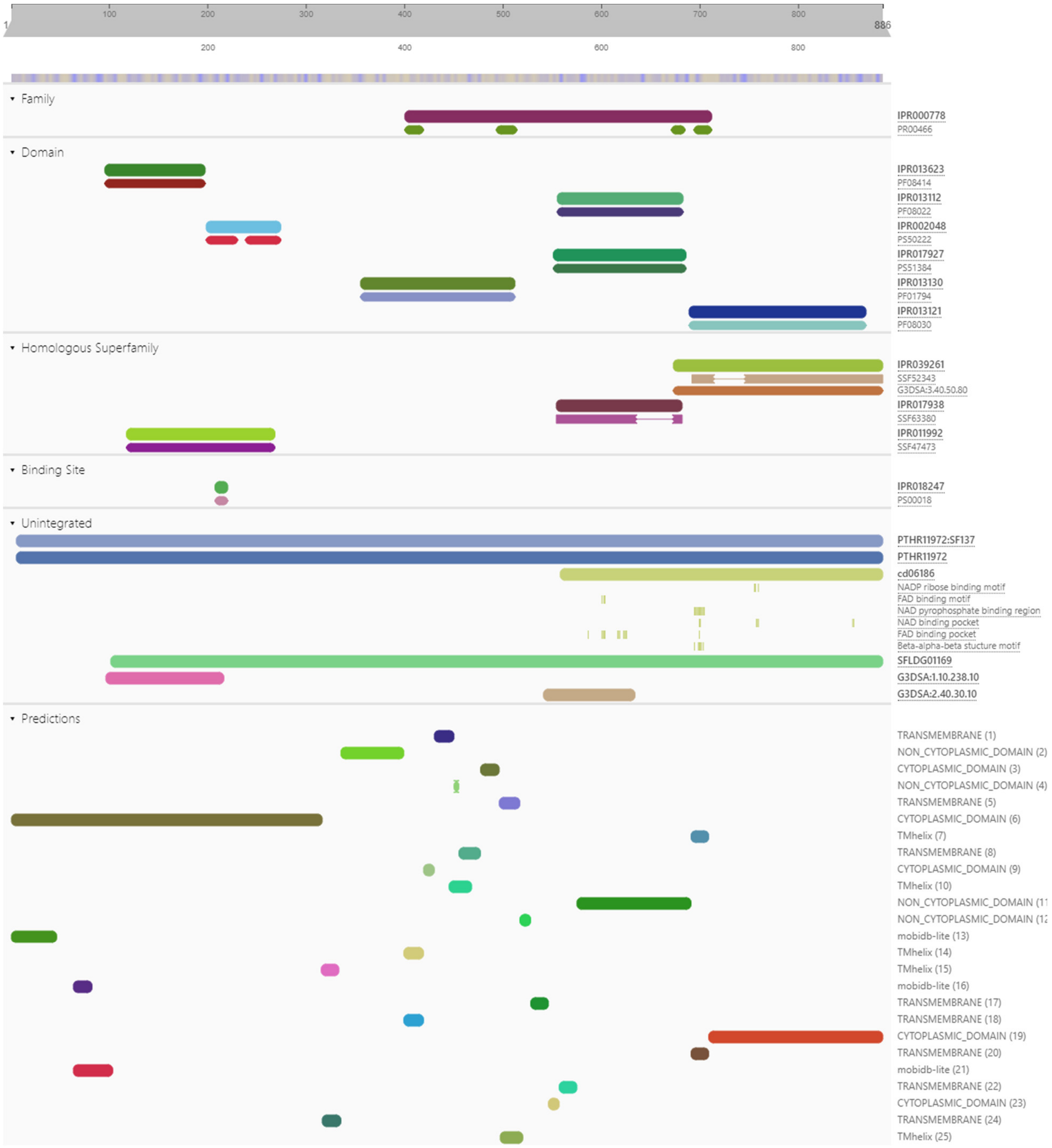

1
100
200
300
400
500
600
700
800
886
200
400
600
800
Family
IPR000778
PR00466
Domain
IPR013623
PF08414
IPR013112
PF08022
IPR002048
PS50222
IPR017927
PS51384
IPR013130
PF01794
IPR013121
PF08030
Homologous Superfamily
IPR039261
SSF52343
G3DSA:3.40.50.80
IPR017938
SSF63380
IPR011992
SSF47473
Binding Site
IPR018247
PS00018
Unintegrated
PTHR11972:SF137
PTHR11972
cd06186
NADP ribose binding motif
FAD binding motif
NAD pyrophosphate binding region
NAD binding pocket
FAD binding pocket
Beta-alpha-beta stucture motif
SFLDG01169
G3DSA:1.10.238.10
G3DSA:2.40.30.10
Predictions
TRANSMEMBRANE (1)
NON_CYTOPLASMIC_DOMAIN (2)
CYTOPLASMIC_DOMAIN (3)
NON_CYTOPLASMIC_DOMAIN (4)
TRANSMEMBRANE (5)
CYTOPLASMIC_DOMAIN (6)
TMhelix (7)
TRANSMEMBRANE (8)
CYTOPLASMIC_DOMAIN (9)
TMhelix (10)
mobidb-lite (13)
TMhelix (14)
TMhelix (15)
mobidb-lite (16)
TRANSMEMBRANE (17)
TRANSMEMBRANE (18)
CYTOPLASMIC_DOMAIN (19)
TRANSMEMBRANE (20)
mobidb-lite (21)
TRANSMEMBRANE (22)
CYTOPLASMIC_DOMAIN (23)
TRANSMEMBRANE (24)
TMhelix (25)

AtRbohE

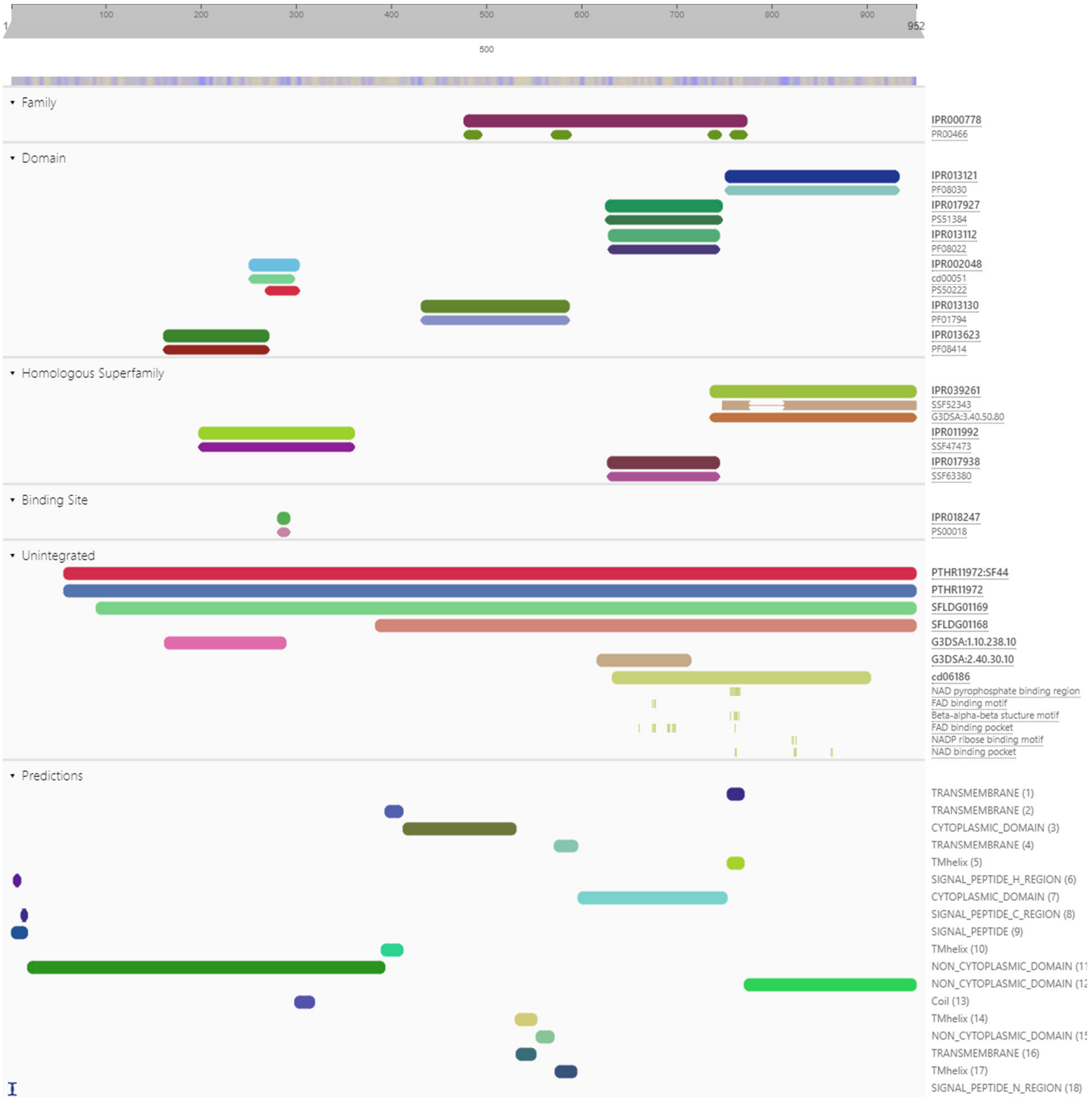

1
100
200
300
400
500
600
700
800
900
952
500
Family
IPR000778
PR00466
Domain
IPR013121
PF08030
IPR017927
PS51384
IPR013112
PF08022
IPR002048
cd00051
PS50222
IPR013130
PF01794
IPR013623
PF08414
Homologous Superfamily
IPR039261
SSF52343
G3DSA:3.40.50.80
IPR011992
SSF47473
IPR017938
SSF63380
Binding Site
IPR018247
PS00018
Unintegrated
PTHR11972:SF44
PTHR11972
SFLDG01169
SFLDG01168
G3DSA:1.10.238.10
G3DSA:2.40.30.10
cd06186
NAD pyrophosphate binding region
FAD binding motif
Beta-alpha-beta stucture motif
FAD binding pocket
NADP ribose binding motif
NAD binding pocket
Predictions
TRANSMEMBRANE (1)
TRANSMEMBRANE (2)
CYTOPLASMIC_DOMAIN (3)
TRANSMEMBRANE (4)
TMhelix (5)
SIGNAL_PEPTIDE_H_REGION (6)
CYTOPLASMIC_DOMAIN (7)
SIGNAL_PEPTIDE_C_REGION (8)
SIGNAL_PEPTIDE (9)
TMhelix (10)
Coil (13)
TMhelix (14)
TRANSMEMBRANE (16)
TMhelix (17)
SIGNAL_PEPTIDE_N_REGION (18)

AtRbohF

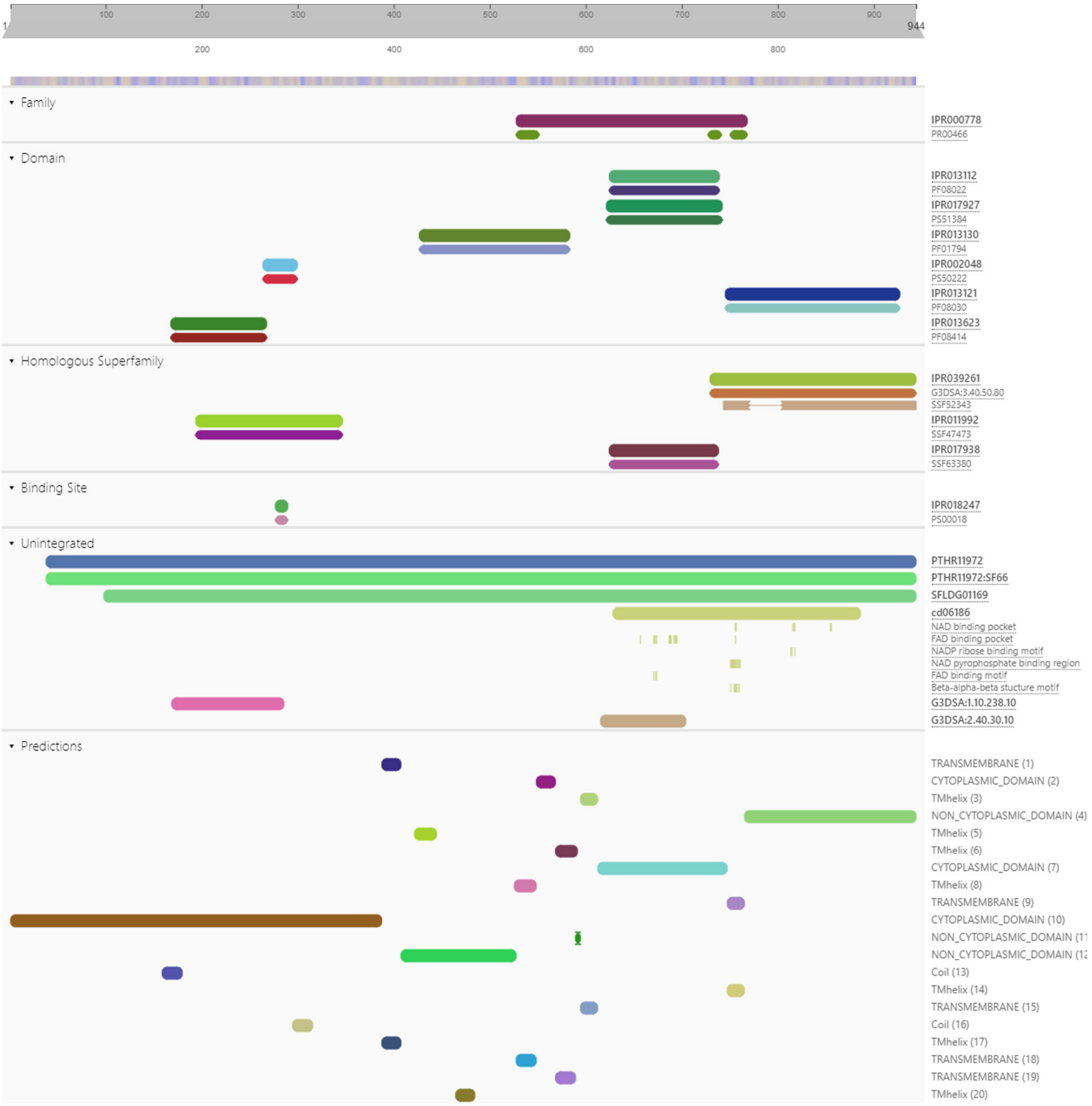
1
100
200
300
400
500
600
700
800
900
944
200
400
600
800
Family
IPR000778
PR00466
Domain
IPR013112
PF08022
IPR017927
PS51384
IPR013130
PF01794
IPR002048
PS50222
IPR013121
PF08030
IPR013623
PF08414
Homologous Superfamily
IPR039261
G3DSA:3.40.50.80
SSF52343
IPR011992
SSF47473
IPR017938
SSF63380
Binding Site
IPR018247
PS00018
Unintegrated
PTHR11972
PTHR11972:SF66
SFLDG01169
cd06186
NAD binding pocket
FAD binding pocket
NADP ribose binding motif
NAD pyrophosphate binding region
FAD binding motif
Beta-alpha-beta stucture motif
G3DSA:1.10.238.10
G3DSA:2.40.30.10
Predictions
TRANSMEMBRANE (1)
CYTOPLASMIC_DOMAIN (2)
TMhelix (3)
NON_CYTOPLASMIC_DOMAIN (4)
TMhelix (5)
TMhelix (6)
CYTOPLASMIC_DOMAIN (7)
TMhelix (8)
TRANSMEMBRANE (9)
CYTOPLASMIC_DOMAIN (10)
Coil (13)
TMhelix (14)
TRANSMEMBRANE (15)
Coil (16)
TMhelix (17)
TRANSMEMBRANE (18)
TRANSMEMBRANE (19)
TMhelix (20)

AtRbohI

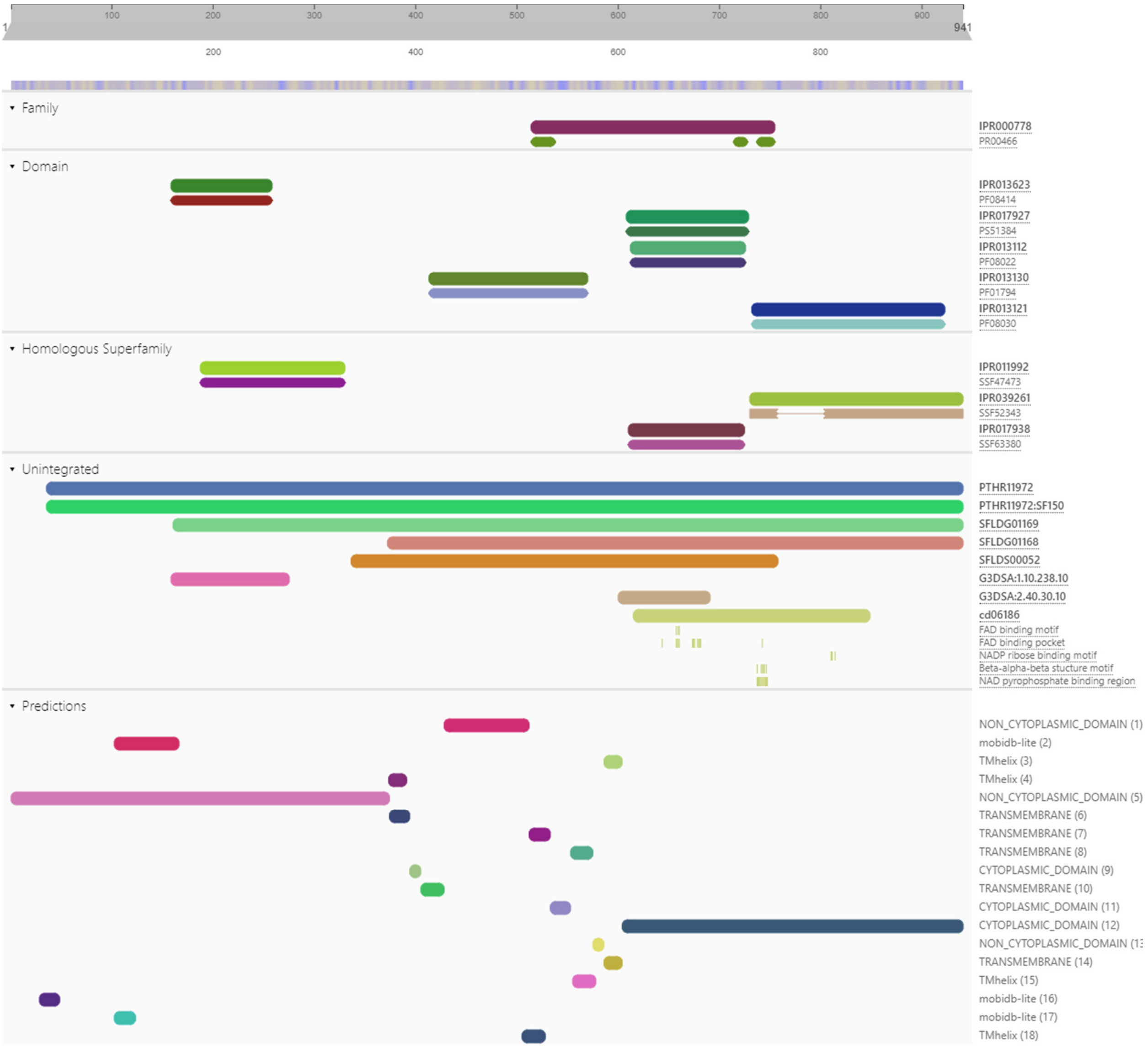

Figure S3. Description of predicted domains in Arabidopsis Rboh proteins.

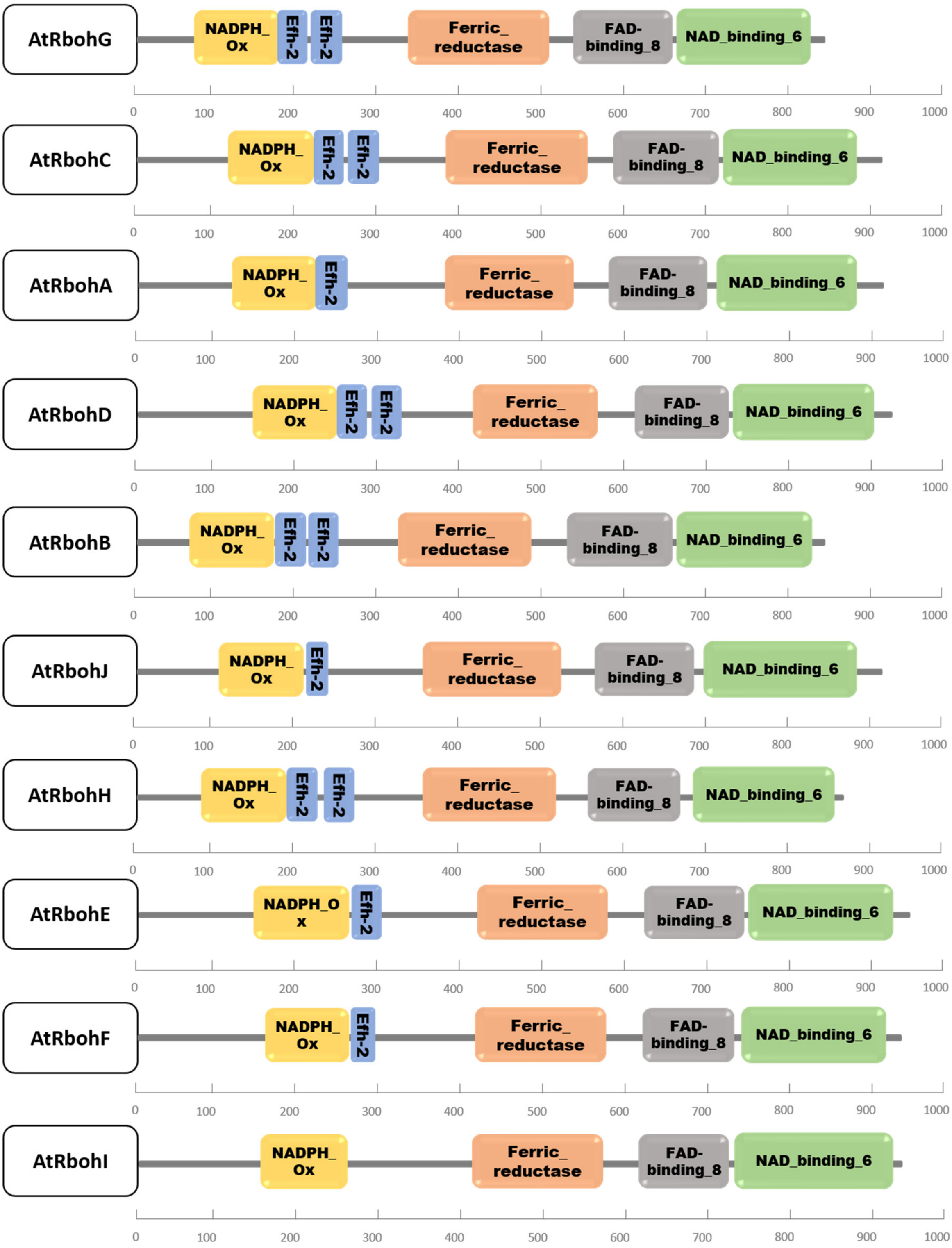

Figure S4. Conserved domains predicted in the Arabidopsis Rboh proteins. Scale below each sequence shows polypeptide length in amino acids. Blocks of different colors represent different domains as well as the EF_hand-2 motif. Width of each block directly correlates with the length of predicted domain in amino acids. X-axis shows the number of amino acids.

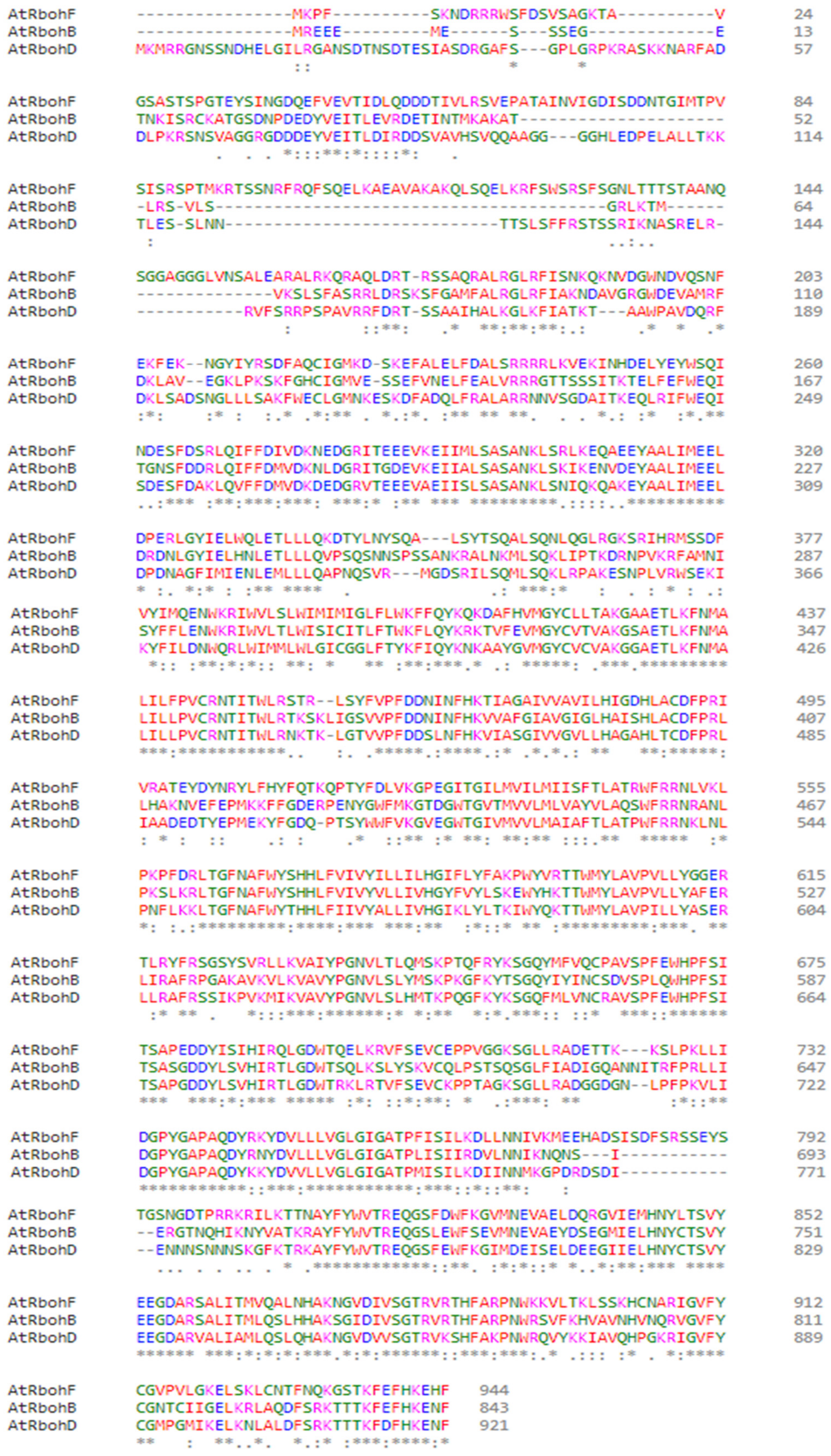

Figure S5. Alignment of RbohB, RbohD and RbohF protein sequences.

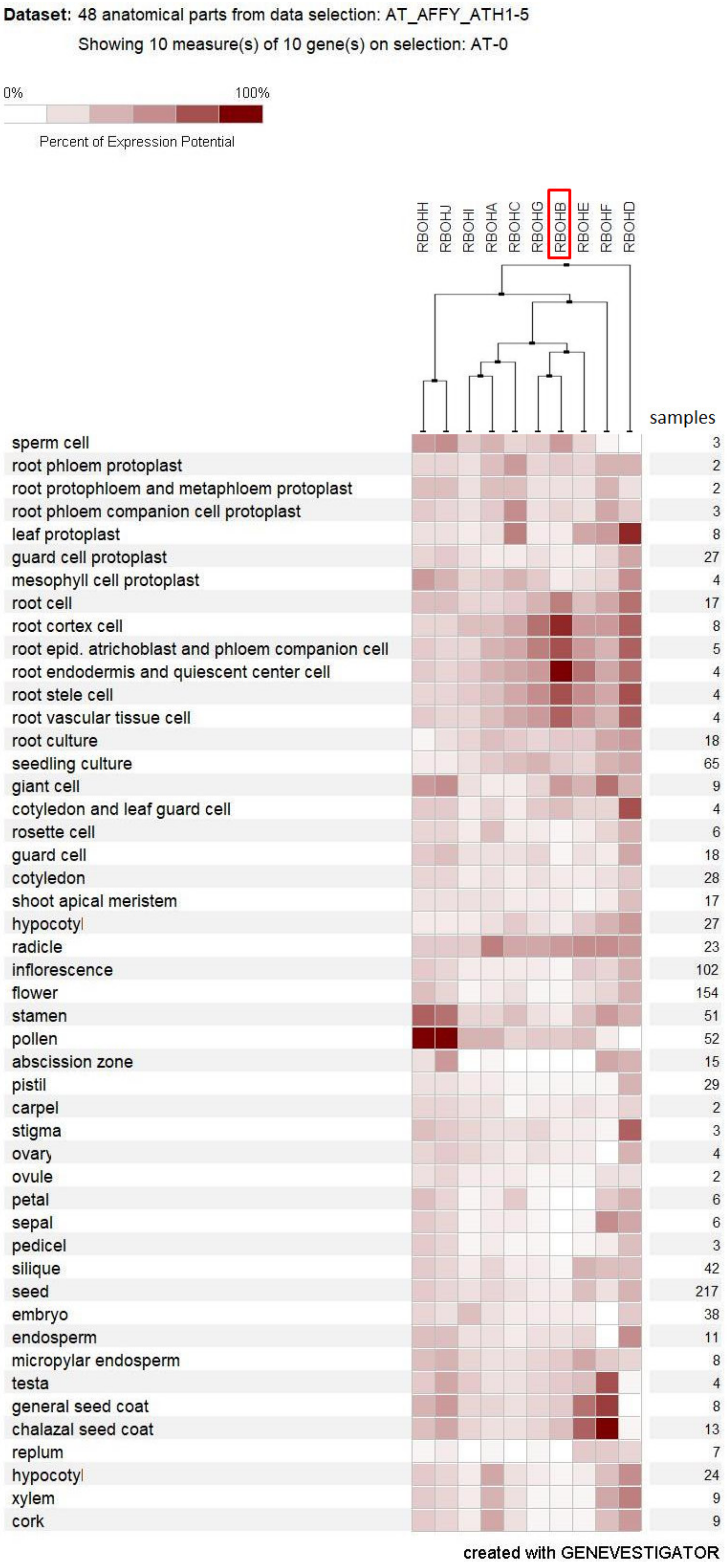

Figure S6. Expression of Arabidopsis Rboh genes in anatomical parts according to Genevestigator.

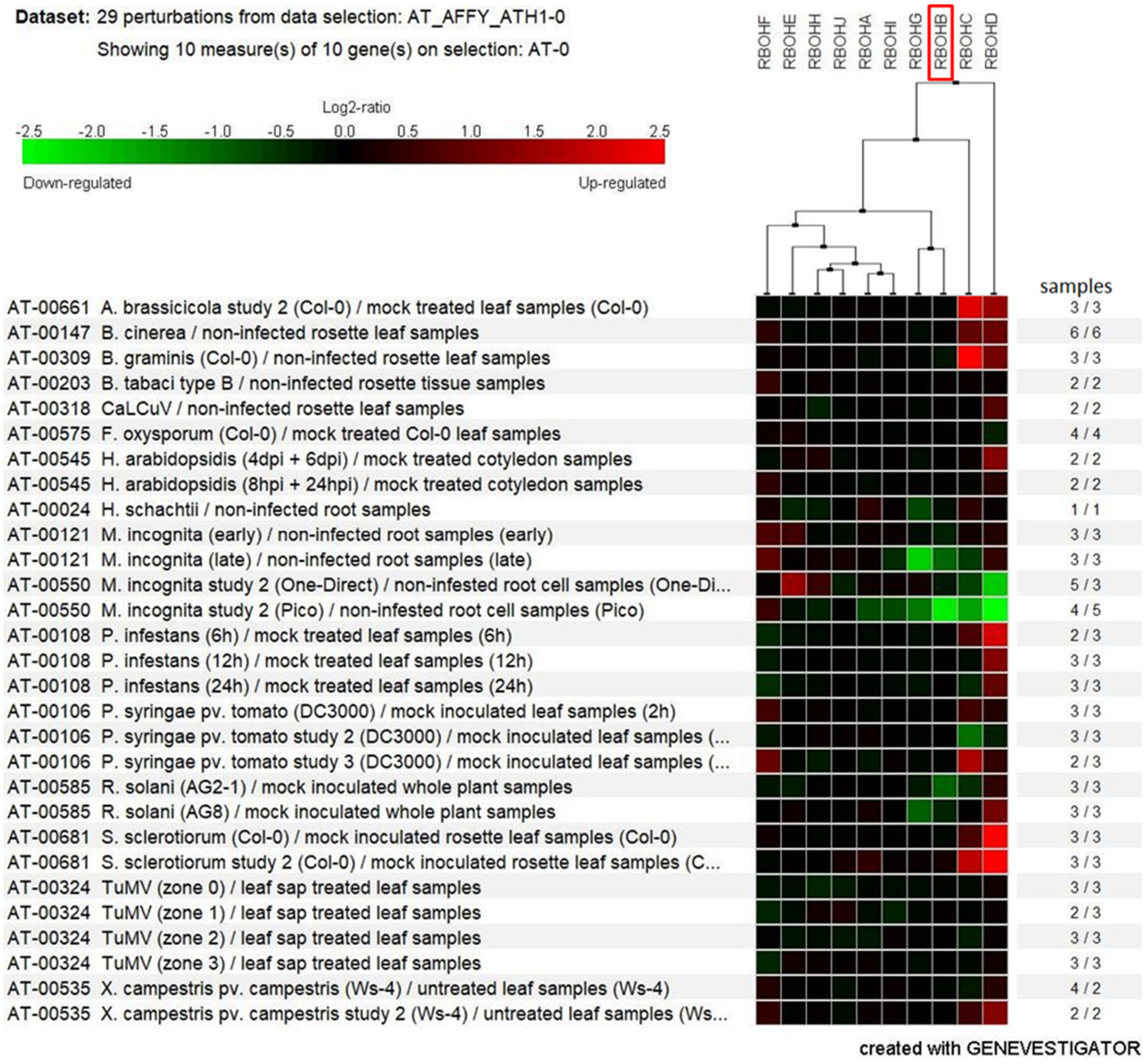

Figure S7. Expression of Arabidopsis Rboh genes after infection with pathogens according to Genevestigator.

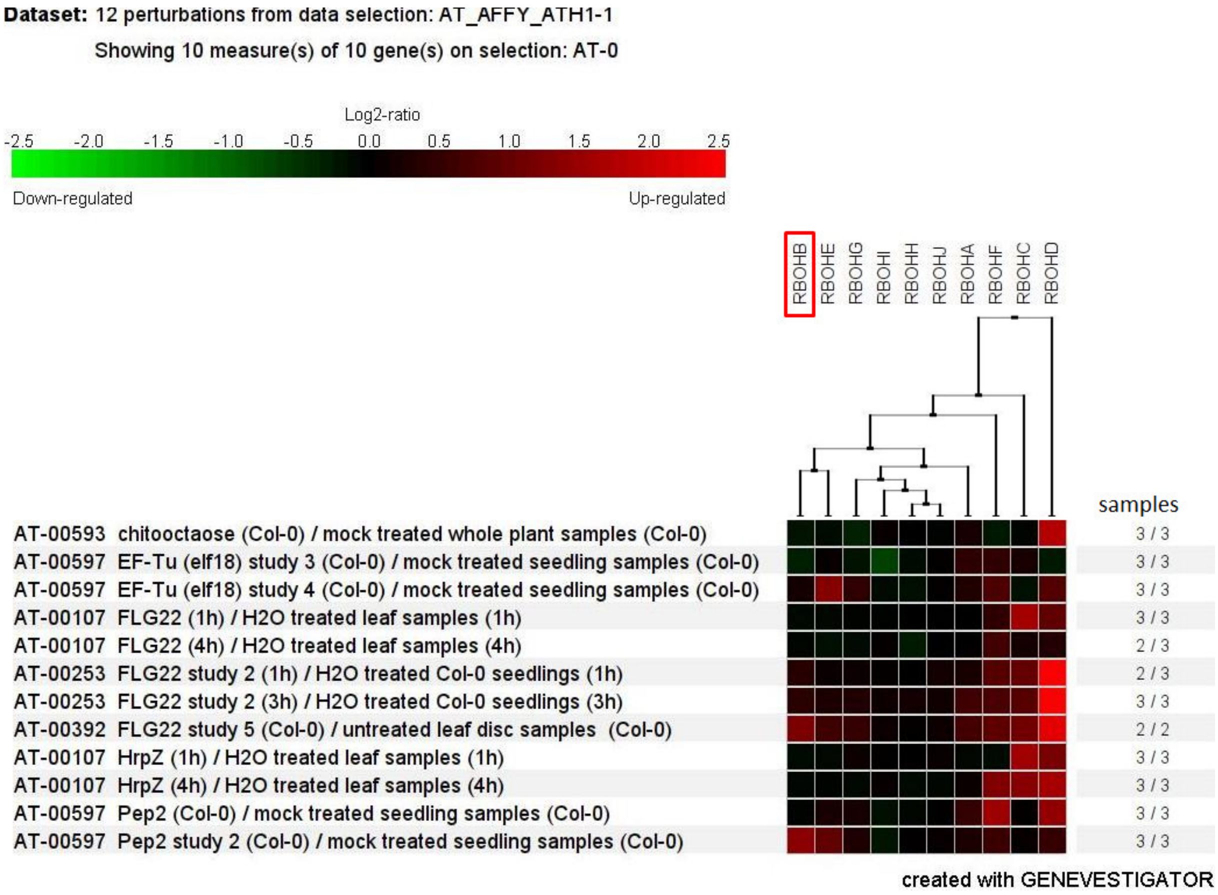

Figure S8. Expression of Arabidopsis Rboh genes after treatment with elicitors according to Genevestigator.

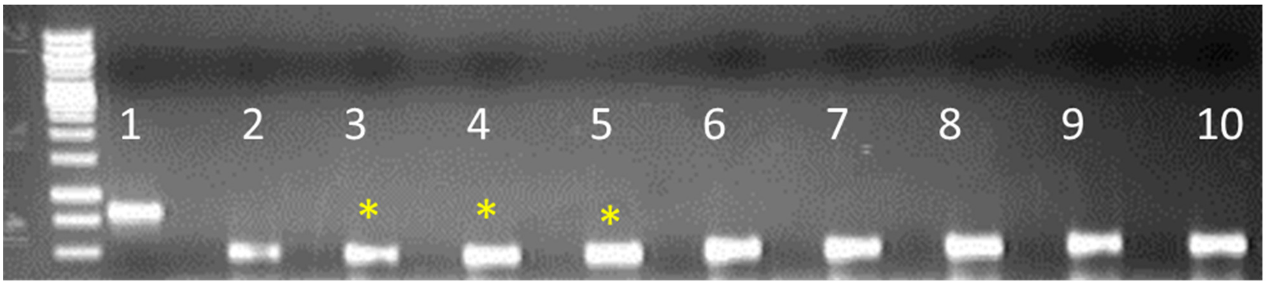

Figure S9. RT-PCR of overexpression lines of RbohB, where 1= gDNA WT, 2=cDNA of WT, 3=cDNA from overexpression line (OE) 1, 4 = cDNA from OE2, 5= cDNA from OE3, 6= cDNA from OE4, 7= cDNA from OE5, 8= cDNA from OE6, 9= cDNA from OE7, 10= cDNA from OE8. cDNA concentration for all the lines was equalized through the measurement of DNA concentrations using Nanodrop. Asterisks show the lines used for characterization. .

© 2020 by the author. Licensee MDPI, Basel, Switzerland. This article is an open access article distributed under the terms and conditions of the Creative Commons Attribution (CC BY) license (http://creativecommons.org/licenses/by/4.0/).
